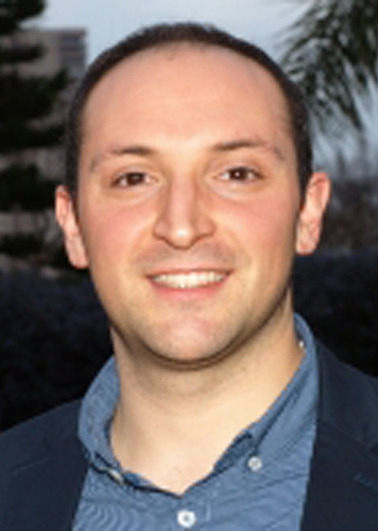# Sustainable Wacker‐Type Oxidations

**DOI:** 10.1002/anie.202211016

**Published:** 2022-10-26

**Authors:** Purushothaman Rajeshwaran, Jonathan Trouvé, Khalil Youssef, Rafael Gramage‐Doria

**Affiliations:** ^1^ Univ Rennes CNRS, ISCR—UMR 6226 F-35000 Rennes France

**Keywords:** Aldehydes, Green Chemistry, Ketones, Olefins, Oxidation

## Abstract

The Wacker reaction is the oxidation of olefins to ketones and typically requires expensive and scarce palladium catalysts in the presence of an additional copper co‐catalyst under harsh conditions (acidic media, high pressure of air/dioxygen, elevated temperatures). Such a transformation is relevant for industry, as shown by the synthesis of acetaldehyde from ethylene as well as for fine‐chemicals, because of the versatility of a carbonyl group placed at specific positions. In this regard, many contributions have focused on controlling the chemo‐ and regioselectivity of the olefin oxidation by means of well‐defined palladium catalysts under different sets of reaction conditions. However, the development of Wacker‐type processes that avoid the use of palladium catalysts has just emerged in the last few years, thereby paving the way for the generation of more sustainable procedures, including milder reaction conditions and green chemistry technologies. In this Minireview, we discuss the development of new catalytic processes that utilize more benign catalysts and sustainable reaction conditions.

## Graphical abstract

Wacker‐type oxidations afford highly value‐added carbonyl compounds starting from readily available olefins and using scarce and expensive palladium catalysts at high dioxygen pressures and elevated temperatures. The last few years have witnessed a paradigm shift in the development of procedures that operate under mild conditions, as discussed in this Minireview. These procedures are based on more abundant and affordable, first‐row transition metals, including metal‐free processes.

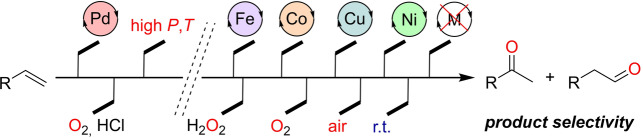

## 1 Introduction

**Scheme 1:**
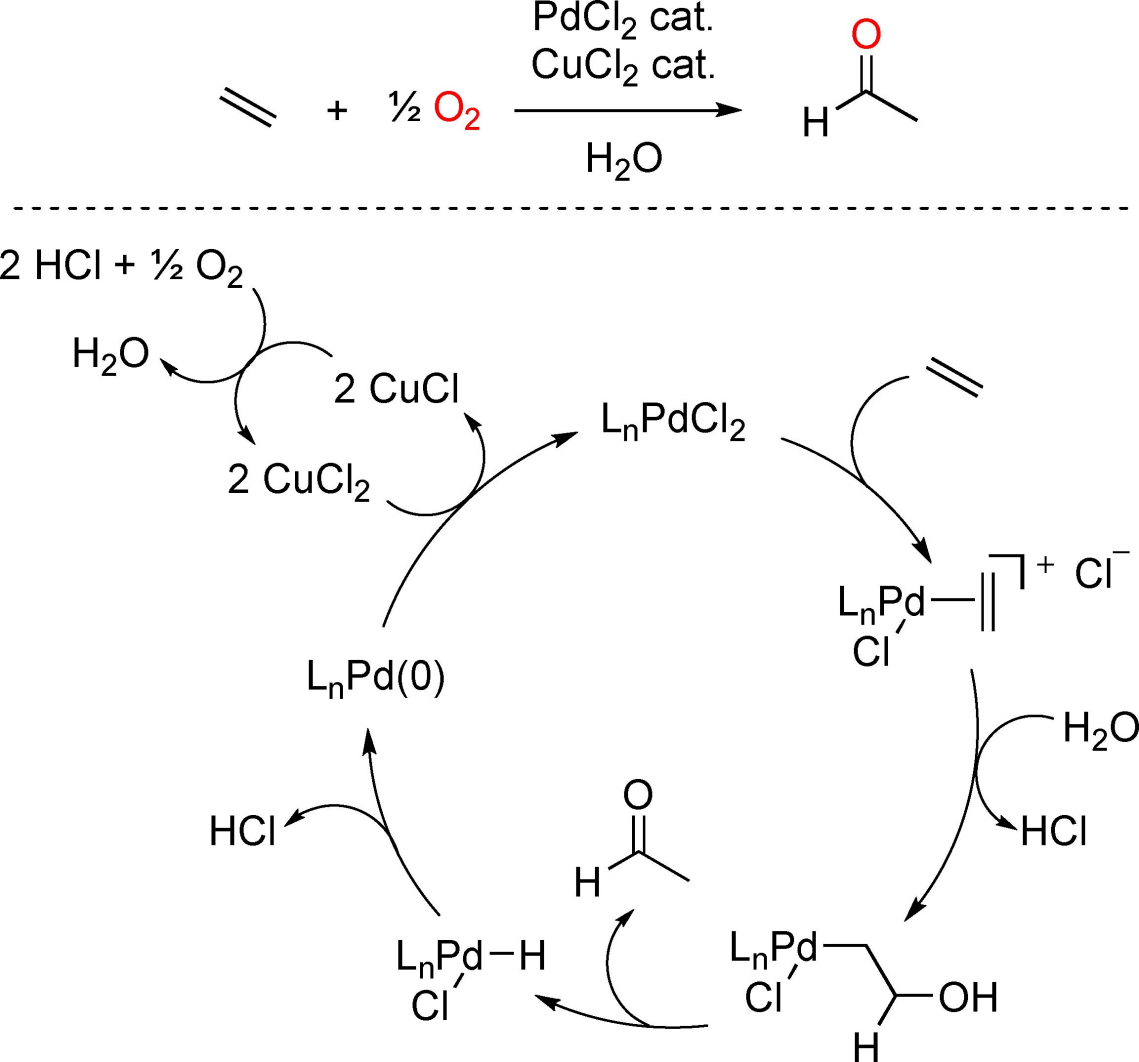
Hoechst–Wacker process (acetaldehyde formation from ethylene, top) and its simplified reaction mechanism (bottom).

The sustainable production of chemicals of daily life is a major challenge for the evolution of humankind.^[1]^ In this regard, oxidation methods starting from raw chemicals provide highly functionalized building blocks in an efficient manner.^[2]^ In particular, access to carbonyl‐containing compounds—either ketones or aldehydes—is crucial, as they are transformed in a straightforward manner into complex materials and active pharmaceutical ingredients, just to mention a few applications.^[3]^ For example, carbonyl compounds are typically prepared in the bulk industry as well as for the preparation of fine‐chemicals for the fragrance industry by means of transition metal‐catalyzed carbonylations of alkenes and alkynes.^[4]^ Alternatively, carbonyl‐containing compounds can be obtained from oxidation processes without increasing the number of carbon atoms in the starting material, in contrast to carbonylations. In this context, Wacker‐type oxidations occupy a central place.^[5]^ Since its discovery in the late 1950s by Smidt et al., and the later implementation at the industrial scale by the Wacker Chemie company,^[6]^ this transformation has found a wide range of applications, ranging from natural product synthesis to the preparation of pharmaceuticals and commodity chemicals. This reaction is highly relevant, since millions of tons of acetaldehyde, a key precursor in a myriad of chemical processes, are prepared by the oxidation of ethylene through a Hoechst–Wacker process (Scheme 1, top).^[7]^

**Figure 1:**
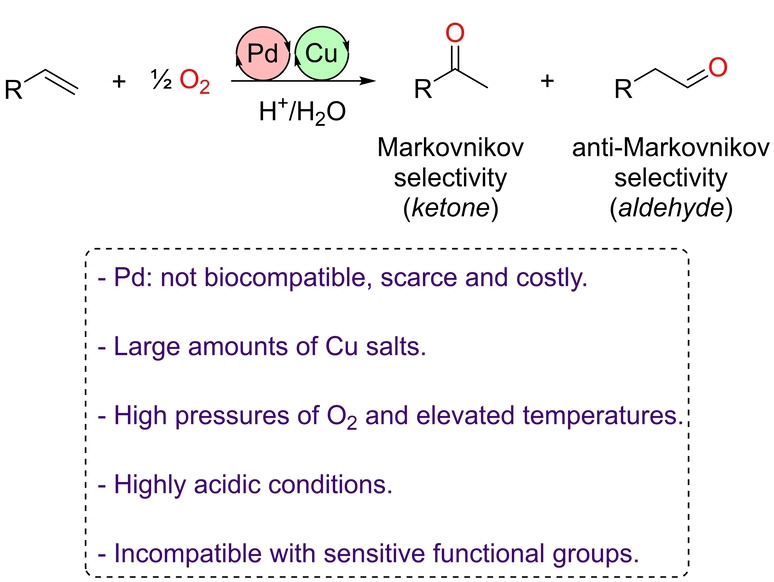
General palladium‐catalyzed Wacker‐type oxidation reaction of olefins and challenges associated with developing greener approaches (framed).

In the broad sense, the Wacker oxidation reaction consists of the oxidation of olefins to ketones (or aldehydes) using a palladium(II) catalyst and a copper(II) co‐catalyst under acidic conditions at high pressures of molecular oxygen (O_2_), and follows the reaction mechanism depicted in Scheme 1 (bottom).^[8]^ In the case of terminal olefins, two products can form depending on which carbon bond is oxidized. As such, the control of this regioselectivity can lead to the Markovnikov product (ketone) or the anti‐Markovnikov one (aldehyde, Figure 1). The regioselectivity is typically imposed by the steric and electronic nature of the substrate or tuned by the reaction conditions, including the palladium‐coordinated ligand employed, which can favor a specific reaction coordinate pathway.^[9]^

In addition to the breakthroughs achieved in the selectivity of Wacker‐type reactions as well as the important progress being made to make such reactions more benign and respectful to the environment (decreasing the reaction temperature, avoiding copper salts and chloride ions, using air instead of pure O_2_),^[10]^ a major limitation still concerns the use of palladium complexes as catalysts (Figure 1). In fact, palladium is a precious noble metal that is scarce in the Earth's crust, costly, and not biologically compatible, which is important considering that trace amounts can remain in the products obtained. Consequently, the last few decades have witnessed a strong interest to replace palladium catalysts by those derived from metals in the first row of the periodic table, with the aim of developing sustainable oxidations including metal‐free processes.^[11]^ In addition, decreasing the catalytic loadings as well as providing milder reaction conditions, namely non‐acidic conditions and at atmospheric pressure, may make the Wacker‐type reaction compatible with sensitive functional groups found in challenging substrates. In this Minireview, we summarize in a comprehensive manner the most important achievements accomplished with the aim of evolving the Wacker‐type reaction into a greener process that is ready to face the challenges associated with the 21st century. Since the aim of this Minireview is to focus on atom economy as well as sustainable transformations relevant to the Wacker‐type process, methods involving two‐step sequences^[12]^ or the use of other noble‐metal‐based catalysts instead of palladium are not discussed herein.^[13]^

## 2 Iron‐Based Catalysts for Wacker‐Type Reactions

Much inspired from the oxidase activity encountered in the natural enzymes from the cytochrome family, numerous efforts have been devoted to develop iron catalysts that enable oxidation reactions.^[14]^ These natural enzymes and their genetically modified analogues as well as purely abiological iron complexes are powerful catalysts for the hydroxylation of aliphatic C−H bonds and epoxidation of olefins with high levels of regio‐, enantio‐, and stereocontrol.^[15]^ Interestingly, a couple of contributions noted that traces of aldehydes (one of the products resulting from the Wacker‐type reaction) formed during oxidation studies of olefins with different types of cytochrome P450s as catalysts under specific reaction conditions.^[16]^ Such promiscuous activity encountered with these enzymes indicated that iron complexes could be suitable for application as catalysts in the Wacker‐type reaction. On the other hand, the fact that iron complexes catalyze reactions typical of noble metals (i.e. hydrogenation, hydrogen borrowing, olefin metathesis, polymerization, hydroformylation, C−H bond functionalizations, etc.) made their application in Wacker‐type reactions conceivable.^[17]^

### 2.1 Iron‐Based Catalysts with Anti‐Markovnikov Selectivity

**Scheme 2:**
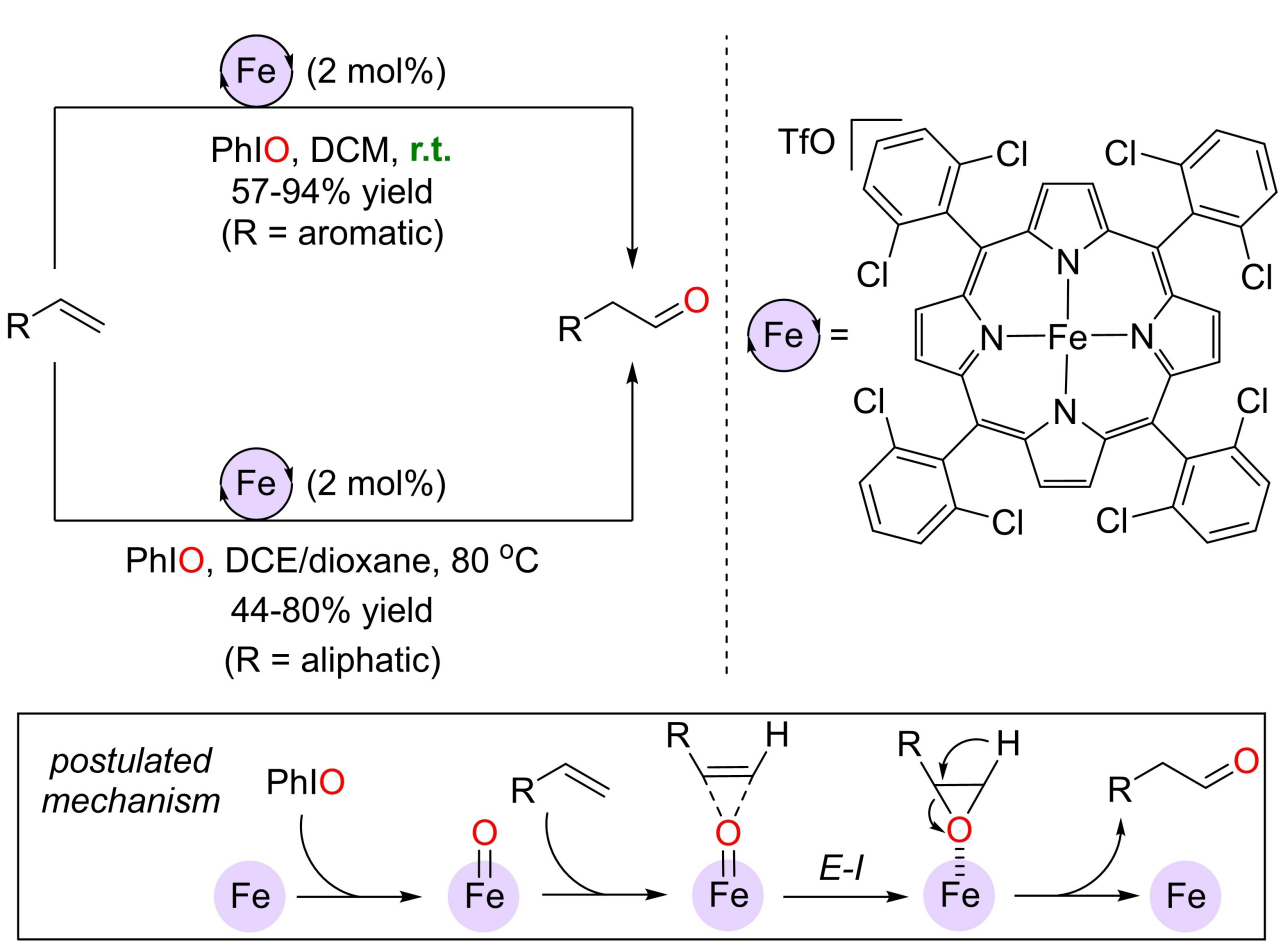
Wacker‐type oxidation of terminal olefins to aldehydes catalyzed by an iron(III) porphyrin with triflate as the anion and its reaction mechanism (framed; the porphyrin backbone around the iron center is not depicted for the sake of clarity). DCM=dichloromethane, DCE=1,2‐dichloroethane, *E*‐*I*=epoxidation‐isomerization.

In 2011, Che and co‐workers reported the first example of a Wacker‐type oxidation of olefins into aldehydes by means of an iron(III) catalyst (Scheme 2).^[18]^ They employed an iron(III) tetraphenylporphyrin featuring 2,6‐dichloro‐substitution patterns in the phenyl groups at the *meso* position and one weakly coordinating ligand CF_3_SO_3_^−^ (TfO^−^), which renders the iron center a stronger Lewis acid. With 2 mol % iron catalyst in the presence of over‐stoichiometric amounts of PhIO oxidant as the oxygen source, a large range of aryl olefins were transformed at room temperature into the corresponding aldehydes without formation of ketone side products. In the case of terminal aliphatic olefins, the reactions required 80 °C when using a mixture of DCE (1,2‐dichloroethane) and dioxane as the solvents. Such high anti‐Markovnikov selectivity was rationalized by the formation of a transient epoxide that undergoes *in situ* isomerization similar to that known for ruthenium(IV) porphyrin catalysts.[^13b^, ^13c^]

**Scheme 3:**
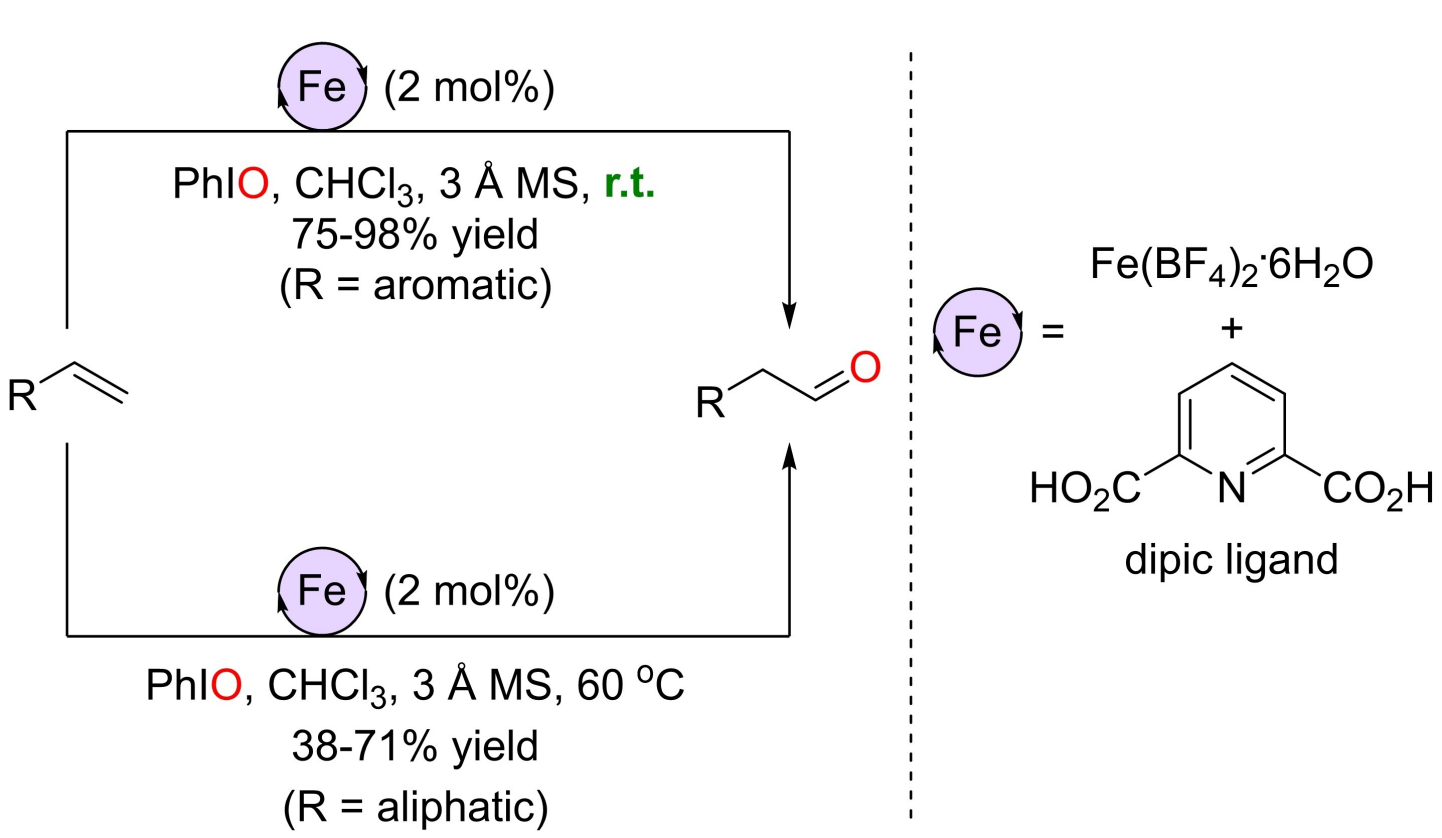
Iron‐catalyzed Wacker‐type oxidation of terminal olefins to aldehydes using dipic as the iron‐coordinating ligand.

Similar reaction conditions, but with chloroform instead of dichloromethane as solvent, were compatible with the formation of aldehydes from terminal olefins when employing a non‐porphyrin iron(III) catalyst (Scheme 3).^[19]^ As in the example above, terminal aliphatic olefins required higher temperatures (60 °C) than the aromatic ones (room temperature). The advantage of this transformation is that it relies on the use of a trivial ligand, namely pyridine‐2,6‐dicarboxylic acid (dipic), and a readily available iron salt such as [Fe(BF_4_)_2_⋅6 H_2_O]. Moreover, the authors noted that the reaction was sensitive to water and required molecular sieves.

**Scheme 4:**
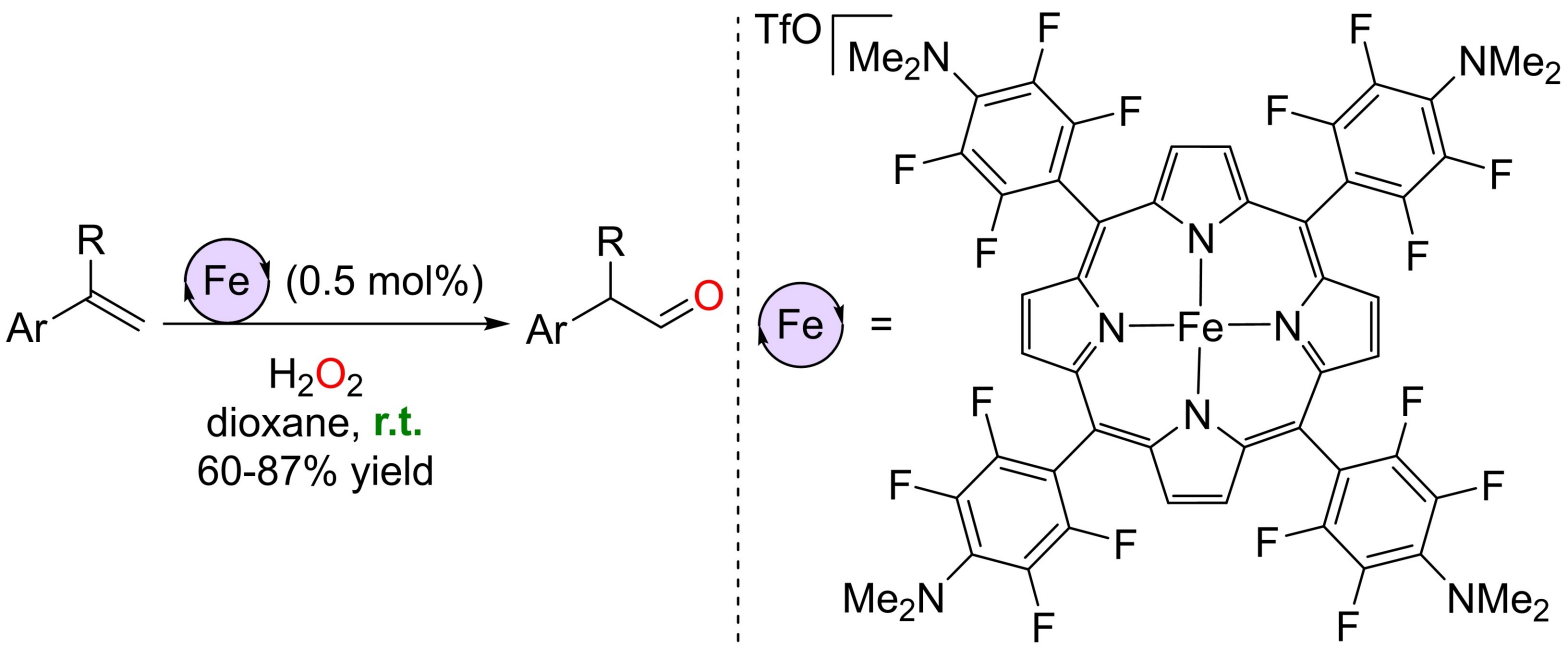
Wacker‐type oxidation of styrene derivatives to aldehydes catalyzed by an iron(III) porphyrin using H_2_O_2_ as the chemical oxidant at room temperature.

The above examples show the possibility of using iron catalysts for the Wacker‐type production of aldehydes from olefins. However, the reactions relied on the use of PhIO, a strongly oxidizing species that needs to be handled with care and generates stoichiometric amounts of PhI or related derivatives as side products, thus limiting the overall atom efficiency. In the search for more convenient oxidizing sources for iron‐catalyzed Wacker‐type reactions, Che and co‐workers reported the ability of a specific iron(III) porphyrin to catalyze this transformation in the presence of H_2_O_2_, which upgrades the level of atom‐economy (Scheme 4).^[20]^ The catalyst was designed in such a way that the corresponding catalytically productive LFe^IV^=O species would be more stable and less oxidizing than state‐of‐the‐art systems because of the electron‐donating character of the tetrafluoro‐*para*‐dimethylanilino functionalities located at the *meso* positions of the porphyrin. This catalyst significantly avoids over‐oxidation side‐reaction pathways at room temperature, thereby leading to full conversion of styrene derivatives and excellent yields of the corresponding aldehydes. Although no examples were reported for aliphatic olefins, the catalyst loading was reduced to 0.5 mol % compared to the 2 mol % in previous examples.[^18^, ^19^]

**Scheme 5:**
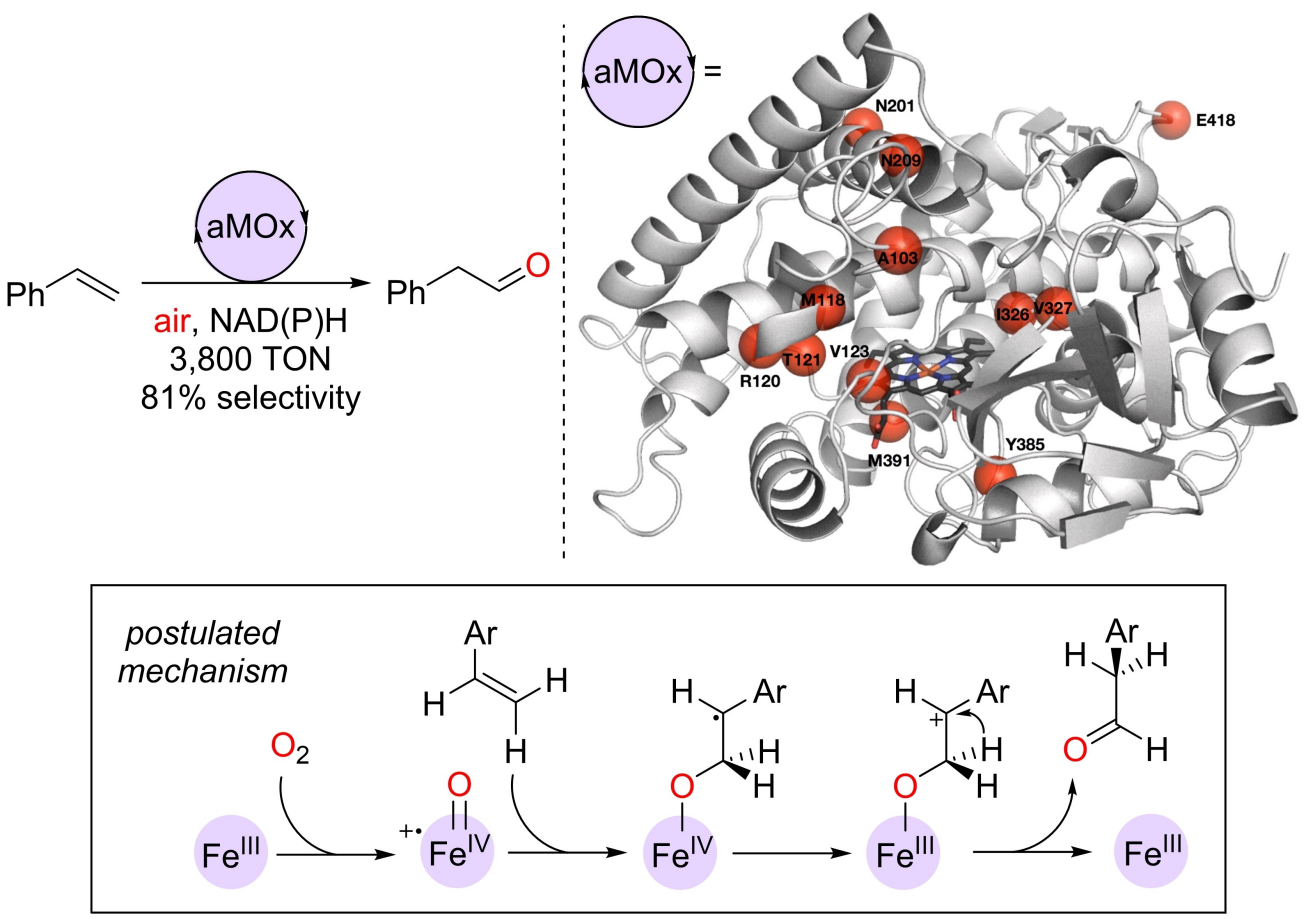
Aerobic anti‐Markovnikov olefin oxidation with the engineered enzyme aMOx found by directed evolution and its postulated reaction mechanism (framed; the heme backbone around the iron center is not depicted for the sake of clarity). NADH=reduced form of NAD^+^ (nicotinamide adenine dinucleotide), NADPH=reduced form of NADP^+^ (NAD^+^ phosphate), TON=turnover number. Scheme adapted from Ref. [21]. Reprinted with permission from AAAS.

**Scheme 6:**
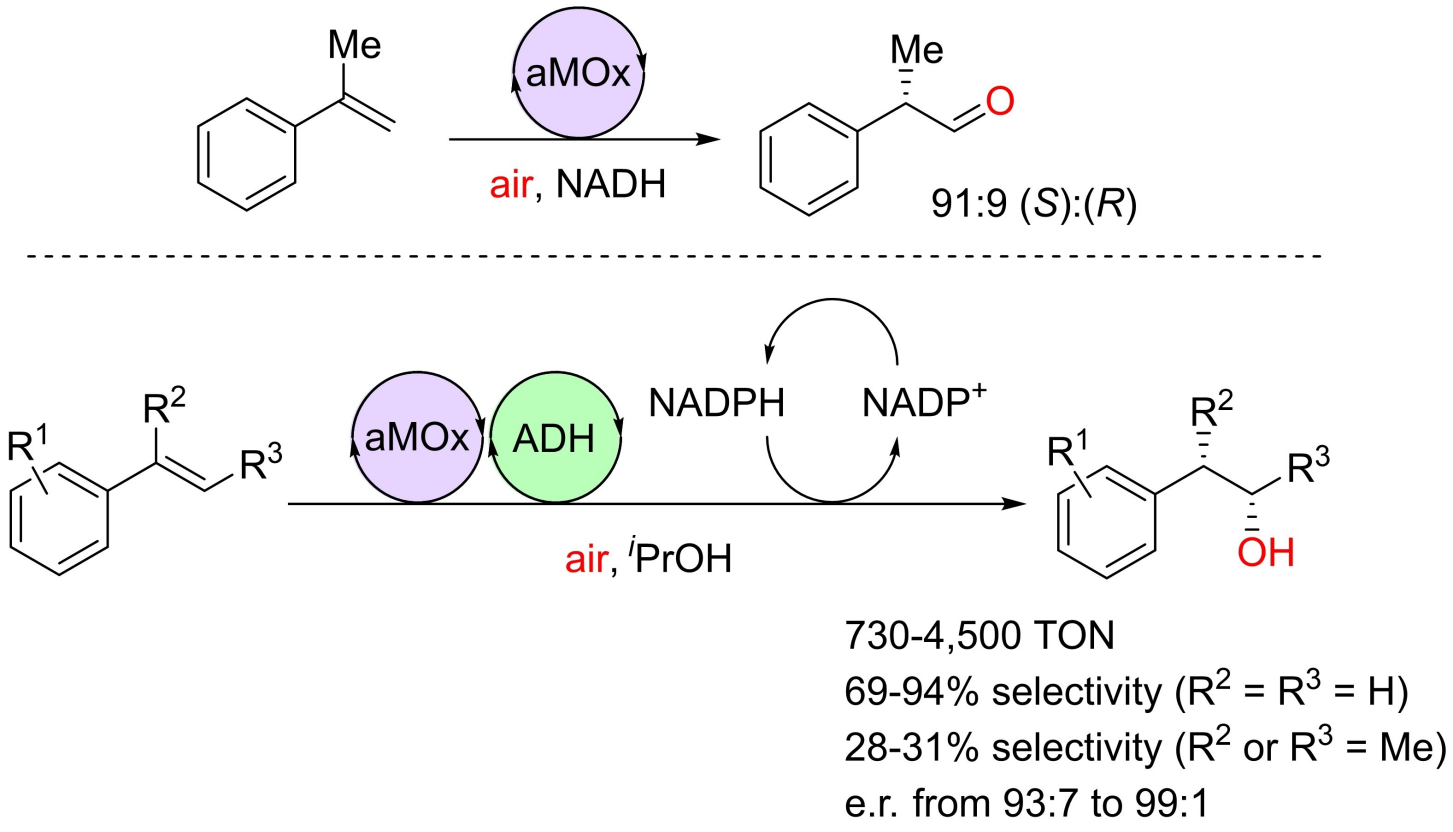
Application of the enzyme aMOx in asymmetric Wacker‐type oxidation catalysis (top) and in combination with an alcohol dehydrogenase (ADH) to biosynthetically afford anti‐Markovnikov chiral alcohol products in a one‐pot fashion under aerobic conditions (bottom). e.r.=enantiomeric ratio.

As it can be noted, both iron(II) and iron(III) complexes are suitable catalysts for the selective anti‐Markovnikov oxidation of olefins to aldehydes provided the active LFe^IV^=O species is formed under the catalytic conditions.[^18^, ^19^, ^20^] Typically, relatively strong oxidizing agents (i.e. PhIO, H_2_O_2_) are required in over‐stoichiometric amounts. Clearly, O_2_ directly from air could be an ideal oxidant for this type of oxidation reaction because of its availability and enhanced compatibility with sensitive functional groups. Such a breakthrough was accomplished in 2017 by the Arnold group, who engineered by directed evolution the enzyme aMOx (anti‐Markovnikov oxygenase), which operates under an ambient pressure of air (Scheme 5).^[21]^ This enzyme, which is derived from the cytochrome P450 family found in the rhodobacterium *Labrenzia aggregata*, contains an iron‐heme active site that catalyzes the oxidation of styrene derivatives to aldehydes with impressive turnover numbers (TONs) above 4000. The standard reaction conditions involved the use of NADH or NADPH as cofactors. It was demonstrated that the catalysis proceeded without the formation of an epoxide intermediate and by an additional electron transfer leading to a high‐energy carbocation intermediate that further undergoes 1,2‐hydride migration, thus generating the anti‐Markovnikov product in the final elementary step (Scheme 5, framed). Recent computational calculations support this mechanism.^[22]^ Moreover, it was possible to control the enantioselectivity of the oxidation by employing a prochiral olefin, which yielded the corresponding aldehyde in an enantiomeric ratio of up to 91 : 9 (Scheme 6).^[21]^ Additionally, the potential of such an approach was exemplified by performing a one‐pot two‐step sequence involving two different enzymes, namely aMOx and an alcohol dehydrogenase (ADH), thereby making it possible to use NADP^+^ in catalytic amounts to formally hydrate alkenes to α‐substituted, enantiomerically pure secondary alcohols (Scheme 6).^[21]^ This study is the first example of a palladium‐free asymmetric Wacker‐type oxidation and it demonstrates the power of biocatalysis to tackle challenging asymmetric reactions.

### 2.2 Iron‐Based Catalysts with Markovnikov Selectivity

**Scheme 7:**
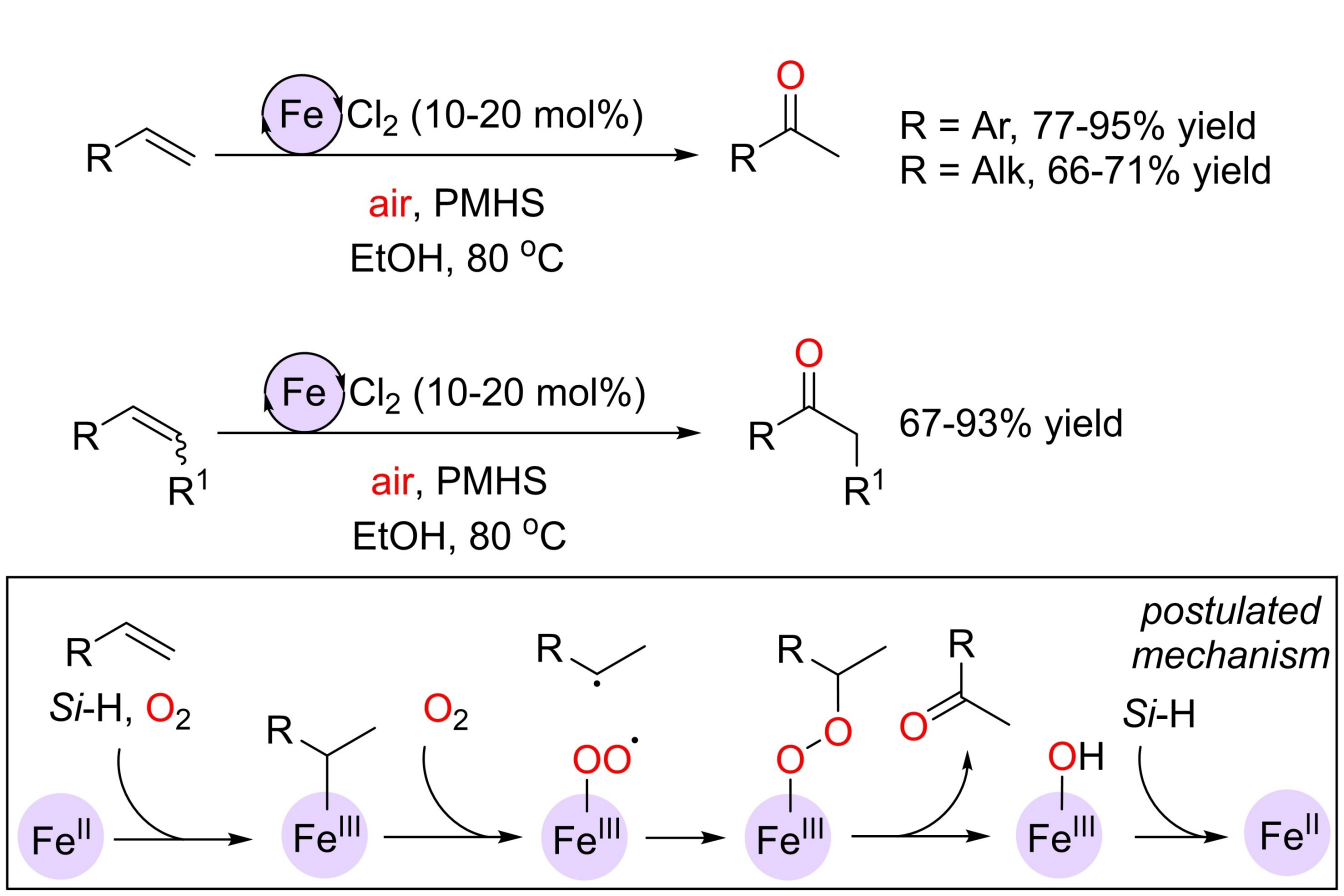
Markovnikov‐selective Wacker‐type iron‐catalyzed oxidation of terminal and internal olefins to ketones with air at room temperature and the postulated reaction mechanism (framed; the ligands around the iron center are not depicted). *Si*‐H=PMHS or other hydrosilane reducing agent.

In addition to the use of iron catalysts to enable anti‐Markovnikov selectivity for terminal olefins, important achievements have been accomplished for developing iron‐catalyzed processes that deliver Markovnikov products, namely ketones. The pioneering contribution in this respect is ascribed to the Han group.^[23]^ In 2017, they reported the oxidation of olefins to ketones with FeCl_2_ (10–20 mol %) as a precatalyst in the presence of over‐stoichiometric quantities of PMHS (PMHS=polymethylhydrosiloxane) as the reducing agent at 80 °C in ethanol solution and in an air atmosphere (Scheme 7). The catalysis was compatible with other types of iron precursors such as Fe(acac)_2_ (acac=acetylacetonate) as well as other hydrosilane reagents [(EtO)_3_SiH] and alcohol solvents (*t*BuOH). Traces of aldehydes resulting from the anti‐Markovnikov selectivity were encountered in some cases. The catalysis was amenable to internal olefins as well, and it tolerated many sensitive functional groups. Several control experiments were conducted to study the reaction mechanism and explain the Markovnikov selectivity. In contrast to the reaction mechanisms leading to anti‐Markovnikov products, which are based on the formation of LFe^IV^=O species, the proposed mechanism here involves an original set of elementary steps (Scheme 7, framed). It starts with the olefin substrate reacting with an *in situ* formed Fe^III^ hydride arising from the combination of an Fe^II^ precursor and the hydrosilane in an O_2_ atmosphere to yield an Fe^III^‐alkyl intermediate that further recombines with O_2_ to afford a carbon‐centered radical intermediate with simultaneous formation of a Fe^III^‐peroxide species. Next, a concerted processes involving both O−O and C−H bond cleavage leads to the ketone product and a Fe^III^‐hydroxide species. The latter undergoes reduction with hydrosilane by regenerating the Fe^II^ active species.

**Scheme 8:**
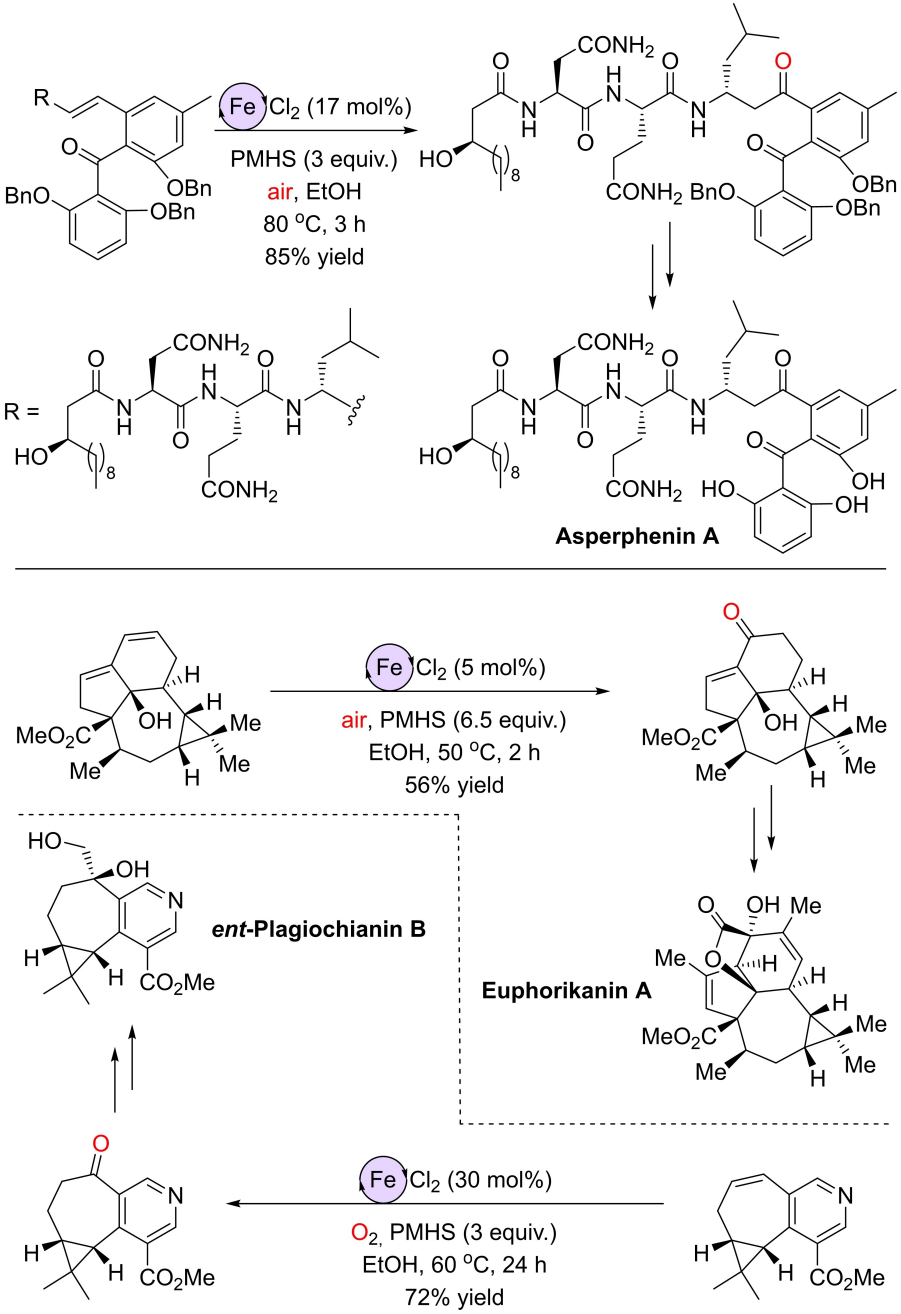
Application of the Markovnikov‐selective Wacker‐type iron‐catalyzed oxidation of olefins to ketones in the total synthesis of Asperphenin A (top), Euphorikanin A (bottom, right), and *ent*‐Plagiochianin B (bottom, left).

The robustness of this method was demonstrated by applying it in the late‐stage functionalization of complex small molecules, thereby establishing its reliability for implementation in total synthesis.^[23]^ Other research groups have implemented the iron‐catalyzed Wacker‐type Markovnikov reaction in the total synthesis of biologically relevant molecules such as Asperphenin A,^[24]^ Euphorikanin A,^[25]^ and *ent*‐Plagiochianin B (Scheme 8).^[26]^ In these examples, the palladium‐catalyzed Wacker oxidation processes were either unselective or unreactive, whereas Han's reaction conditions are milder, selective, and compatible with the many functional groups found in the molecules of interest. Although a high iron catalyst loading was needed, these challenging functionalizations were achieved in excellent yields considering the complexity of the targeted products.

**Scheme 9:**
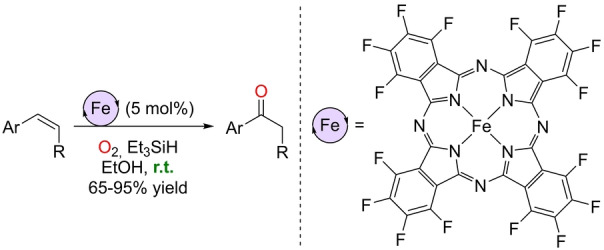
Wacker‐type oxidation of terminal aromatic olefins to ketones catalyzed by an hexadecafluorinated iron(II) phthalocyanine using O_2_ as the oxidant at room temperature.

The development of Wacker‐type iron‐catalyzed oxidations with Markovnikov selectivity witnessed a second breakthrough in 2018 with the contribution by the Knölker group (Scheme 9).^[27]^ They accomplished the oxidation of aromatic olefins to ketones at room temperature and with a relatively low catalyst loading (5 mol %) compared to Han's contribution. Decreasing the temperature from 80 °C to room temperature while keeping the Markovnikov selectivity was possible by using the hexadecafluorinated iron(II) phthalocyanine complex as the precatalyst. The hydrosilane loading was reduced to 2 equivalents by using Et_3_SiH as the reducing agent. In this case, there were no traces of epoxides or aldehydes as side products but of alcohols with the same Markovnikov selectivity. The uniqueness of this iron catalyst was demonstrated by the poor reactivity encountered in the analogous nonfluorinated iron(III) phthalocyanine and perfluorinated iron(III) tetraphenylporphyrin complexes.

**Scheme 10:**
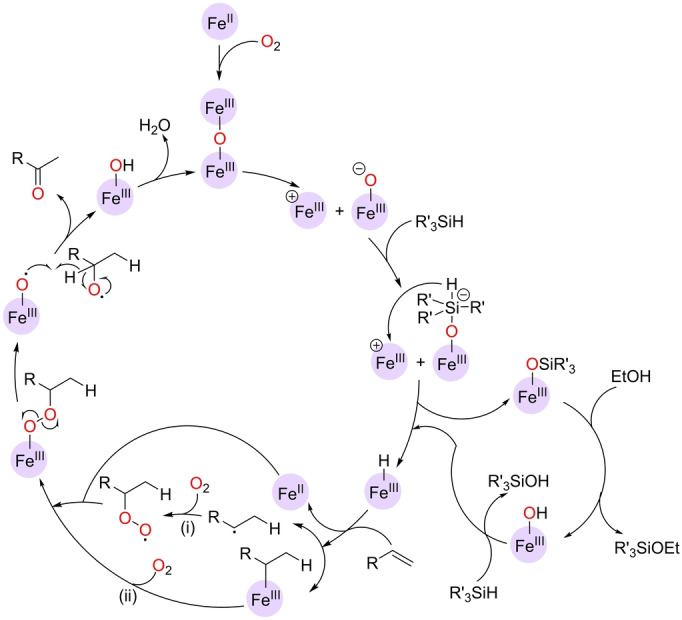
Postulated reaction mechanism for the Wacker‐type oxidation of terminal olefins to ketones catalyzed by a hexadecafluorinated iron(II) phthalocyanine (the phthalocyanine ligand around the iron center is not depicted for the sake of clarity).

The mechanism has been studied experimentally in detail by combining different spectroscopic techniques (Mössbauer spectroscopy, UV/Vis specroscopy, magnetic susceptibility, HRMS, etc.), kinetic studies, and isotopic labeling experiments (with C_2_D_5_OD, Et_3_SiD, and ^18^O_2_).^[28]^ The plausible reaction mechanism is shown in Scheme 10, which establishes the initial formation of a μ‐oxo‐bridged diiron(III) species by oxidation of the iron(II) phthalocyanine precatalyst, which undergoes dissociation towards a cationic Fe^III^ species and an anionic oxido‐Fe^III^ complex. The latter reacts with hydrosilane to generate a pentavalent silicon intermediate that transfers the hydride to the cation Fe^III^ species, thereby forming a postulated Fe^III^‐hydride species. Note that an ethanol‐mediated pathway may also operate to form the Fe^III^‐hydride species, which is likely involved in the rate‐determining step of the catalysis. The Fe^III^‐hydride species could further react with the olefin substrate and O_2_ to generate an alkylperoxy‐Fe^III^ complex by two possible pathways: i) through a “naked” carbon‐centered radical species or ii) through a Fe^III^‐alkyl species (Scheme 10). The final step is the homolytic fragmentation of the alkylperoxy‐Fe^III^ complex and rearrangement towards the ketone product, while the Fe^III^‐hydroxide species regenerates the μ‐oxo‐bridged diiron(III) complex. The formation of alcohol by‐products was rationalized by the presence of side reactions involving ⋅OH radical transfer between the intermediates, Russel fragmentation within peroxide species, or the formal reduction of a ketone to an alcohol with iron‐hydride species.

**Scheme 11:**
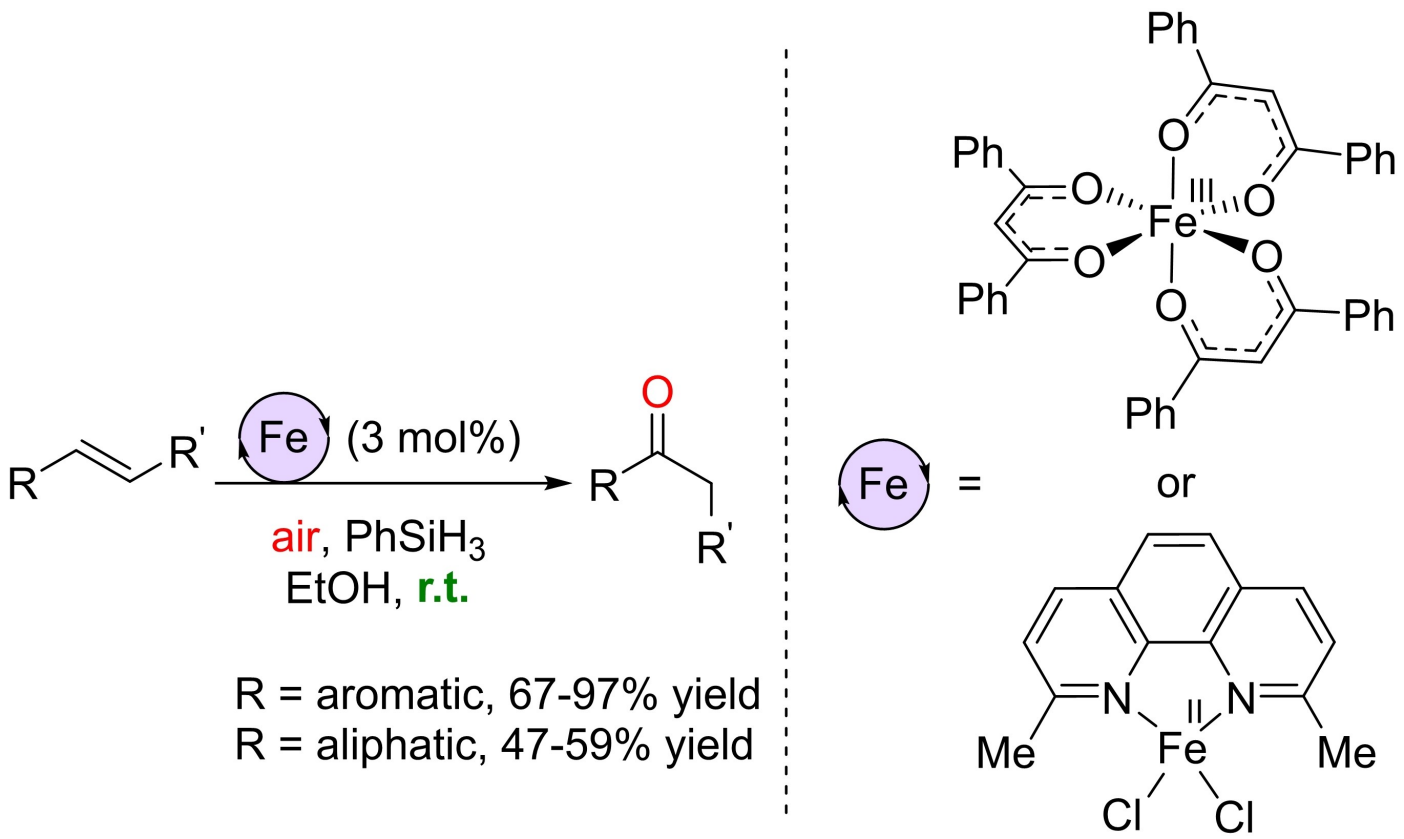
Wacker‐type oxidation of olefins to ketones catalyzed by iron catalysts using air as the oxidant at room temperature.

**Scheme 12:**
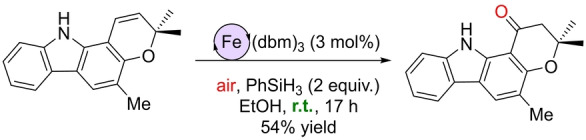
Application of the Markovnikov‐selective Wacker‐type iron‐catalyzed oxidation of olefins to ketones in an air atmosphere at room temperature during the late‐stage functionalization of Girinimbine. dbm=dibenzoylmethanato.

**Scheme 13:**
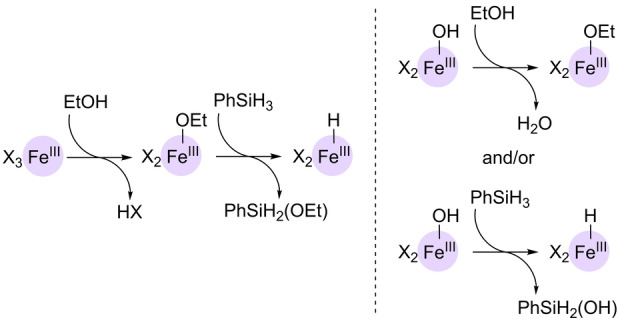
Catalyst formation (left) and catalyst regeneration pathways (right) in the Markovnikov‐selective Wacker‐type oxidation of olefins to ketones catalyzed by iron catalysts using air as the oxidant at room temperature. X=dibenzoylmethanato.

Pushing the boundaries of sustainable Wacker‐type oxidations, the Knölker group reported in 2021 the first examples of iron catalysts operating in an air atmosphere and at room temperature with Markovnikov selectivity.^[29]^ The precatalysts are based on a hexacoordinated iron(III) complex comprising three *O*,*O*‐chelating dibenzoylmethanato (dbm) ligands or an *in situ* combination of FeCl_2_ and the *N*,*N*‐chelating neocuproine ligand (Scheme 11). PhSiH_3_ was the hydrosilane that performed the best and variable amounts of alcohols side products were formed depending on the nature of the substrate. The iron catalysts did not only work for aromatic olefins but also for aliphatic olefins, including internal ones. In addition, its application towards the late‐stage functionalization of Girinimbine, a natural product, was successfully achieved at room temperature at ambient pressure (Scheme 12). Its application in the total synthesis of structurally related Euchrestifoline was also demonstrated.^[30]^ The reaction mechanism that was postulated on the basis of labeling studies established that O_2_ from air was the source of the oxygen and that the hydrosilane delivered the hydrogen atom found in the ketone product. Most of the reaction intermediates are the same as those represented in Scheme 10. The main difference is the formation of the catalytically productive species. This time, a ligand exchange by ethanol at the iron complex may lead to an Fe^III^ ethoxide which is presumed to react with the hydrosilane (PhSiH_3_) to form the key Fe^III^ hydride (Scheme 13, left). The remaining catalytic cycle is the same as depicted in Scheme 10, with the catalyst regeneration occurring by a reaction between the Fe^III^ hydroxide and ethanol to form water and the catalytically active Fe^III^ ethoxide or by reaction with the hydrosilane to form the key Fe^III^ hydride (Scheme 13, right).

**Scheme 14:**
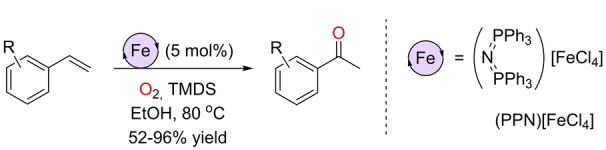
Wacker‐type oxidation of styrenes to ketones catalyzed by an iron(III) ionic catalyst using O_2_ as the oxidant and TMDS at high temperature.

Similarly, Yamaguchi and co‐workers developed an iron‐catalyzed Wacker‐type oxidation of styrenes to ketones by utilizing 5 mol % of a ferrate Fe^III^ complex as precatalyst (Scheme 14).^[31]^ The reaction conditions are similar to those reported independently by the groups of Han and Knölker. They consist of TMDS (TMDS=1,1,3,3‐tetramethyldisiloxane) as the reducing agent in ethanol at 80 °C in an O_2_ atmosphere. This catalytic system is less active than the previous ones, as shown by the lack of reactivity towards internal olefins and aliphatic ones as well as the impossibility to operate in an air atmosphere. Importantly, they noted that FeCl_3_ was not a suitable precatalyst, but it becomes active once it is associated to the cation PPN^+^.

Overall, the above‐stated examples of iron‐catalyzed Wacker‐type processes demonstrate that Fe^III^ species are the active ones and that the role of the Fe^II^ or Fe^III^ precursor complex has a strong effect on the final outcome of the catalysis. In addition, the key role of the Fe^III^‐hydride intermediate has been evidenced, although its characterization remains elusive to date. The manifold reaction mechanisms than can be devised with appropriate ligands engineered at the molecular level for coordinating iron are responsible for the formation of Markovnikov or anti‐Markovnikov oxidation products. It is well‐established that the current state‐of‐the‐art iron catalysts for Wacker‐type oxidations compete, and even outperform in some cases, the most reactive palladium catalysts with regard to the broad functional group tolerance and ease of operability at room temperature and ambient pressure.

## 3 Wacker‐Type Reactions with First‐Row Transition Metal Catalysts Other than Iron

### 3.1 Wacker‐Type Reactions with Cobalt‐Based Catalysts

**Scheme 15:**
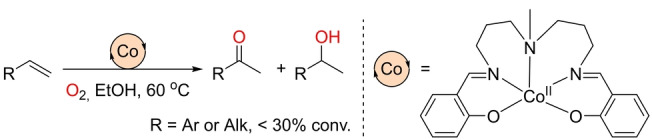
Lack of chemoselectivity found in the Markovnikov‐selective Wacker‐type oxidation of olefins using a cobalt catalyst under O_2_ at high temperature.

In addition to the preponderance of iron catalysts used to replace palladium ones in Wacker‐type oxidations, other base metals from the first row of the periodic table have been studied. In this respect, the pioneering contribution by Drago and co‐workers in the 1980s is noteworthy. They reported a cobalt complex that behaves as a catalyst for the oxidation of terminal olefins in the presence of O_2_ in ethanol solution (Scheme 15).^[32]^ Although the reaction displayed excellent Markovnikov selectivity, almost equimolar amounts of ketone and alcohol were observed with very modest yields of isolated products. This inspired a number of research groups to develop procedures typically based on a two‐step sequence involving first the formation of hydroperoxides followed by an acetylation to generate the ketone.^[33]^ These methods are beyond the scope of this Minireview, since they suffer from low step and atom economy.

**Scheme 16:**
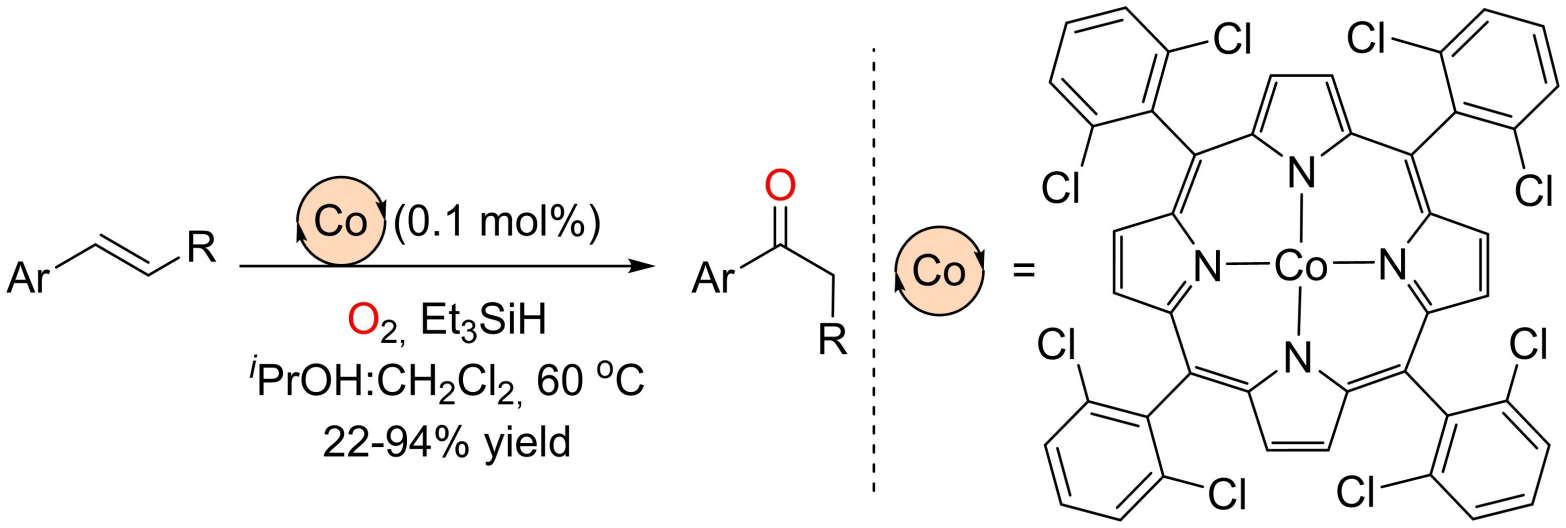
Markovnikov‐selective Wacker‐type oxidation of olefins using a cobalt(II)‐porphyrin catalyst and O_2_ as the oxidant at high temperature.

On the other hand, and similar to the preliminary observations by Drago and co‐workers, the Matsushita group found back in 1992 that a hexachlorinated Co^II^ porphyrin behaved as an excellent catalyst for the Markovnikov‐selective Wacker‐type oxidation of olefins to ketones at a catalyst loading as low as 0.1 mol % (Scheme 16).^[34]^ The reaction conditions were similar to those reported independently by the groups of Han and Knölker for the iron‐catalyzed version with the same regioselectivity. Et_3_SiH was employed as the reducing agent in the presence of an O_2_ atmosphere in a 1 : 1 mixture of 2‐propanol and dichloromethane as the solvents.

**Scheme 17:**
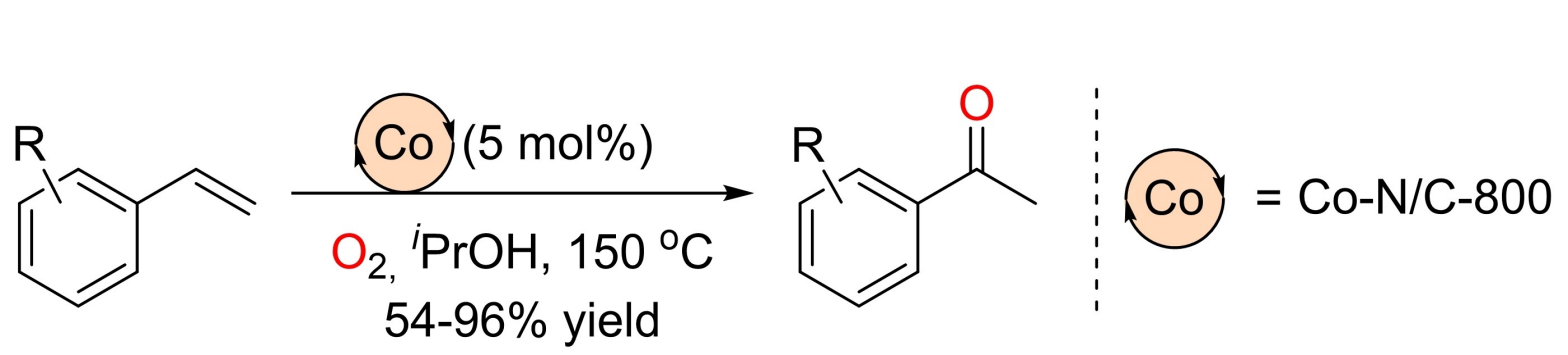
Markovnikov‐selective Wacker‐type oxidation of styrenes to acetophenones catalyzed by a heterogeneous cobalt‐based system under O_2_ pressure at high temperature.

Recently, An and co‐workers reported a heterogeneous cobalt catalyst that enables the Markovnikov‐selective Wacker‐type oxidation of styrenes to acetophenones (Scheme 17).^[35]^ The catalyst was formed upon pyrolysis of mixtures of Co(OAc)_2_ with phenantroline (N) in ethanol supported on activated charcoal (C). The most active and selective heterogeneous catalyst was Co‐N/C‐800 (synthesized by pyrolysis at 800 °C), which afforded the Markovnikov products in 2‐propanol solution. The 2‐propanol is both the hydrogen source of the reaction and the solvent. The catalysis occurs in the absence of any additional reducing agent, but at elevated temperatures (150 °C) and high pressures of O_2_ (40 bar). Monodisperse cobalt atoms appear to be the catalytically active species rather than nanoparticles.

**Scheme 18:**
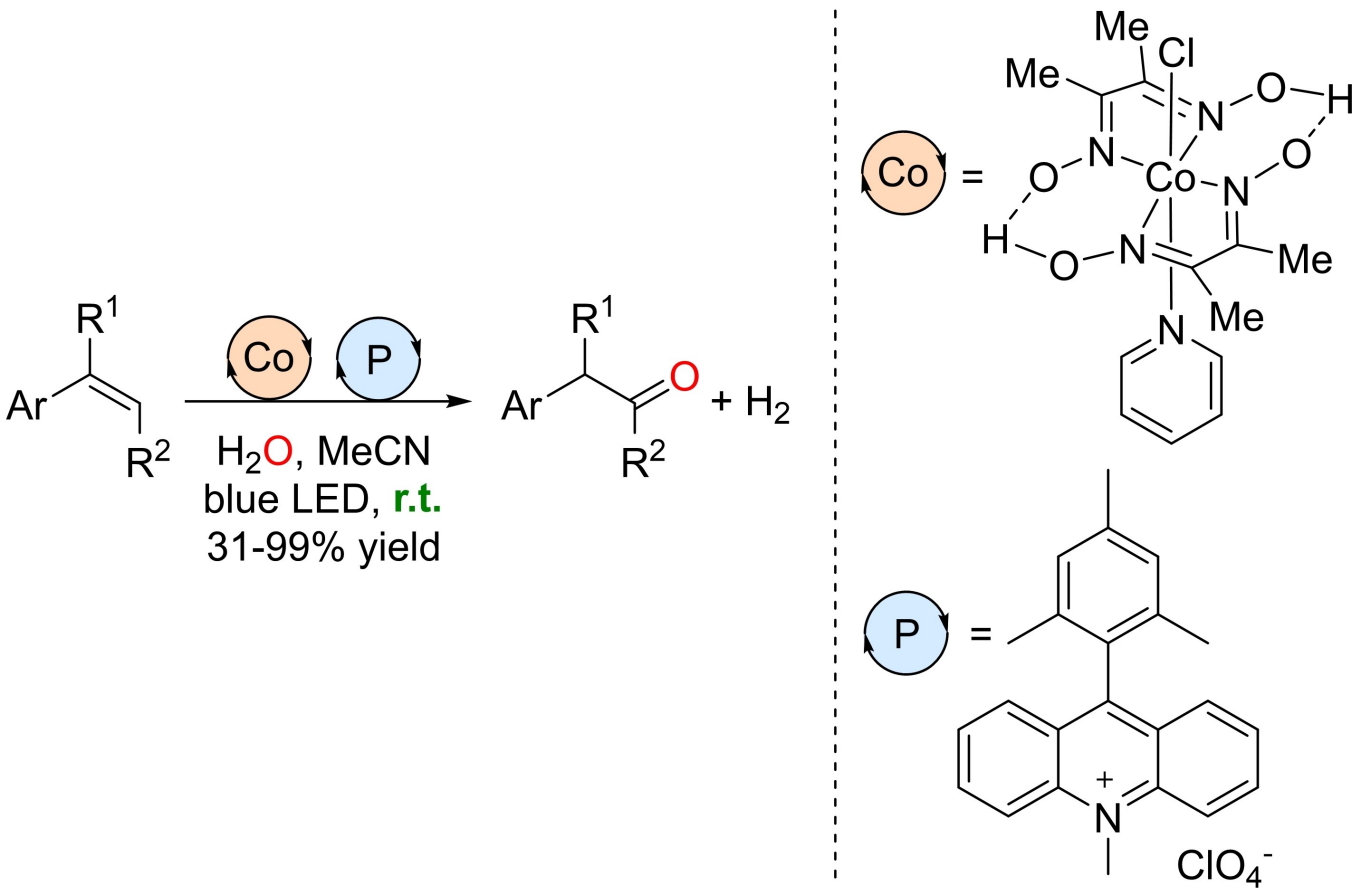
Photocatalytic anti‐Markovnikov Wacker‐type oxidation of β‐alkyl styrenes by water at room temperature with the liberation of dihydrogen. P=photosensitizer.

**Scheme 19:**
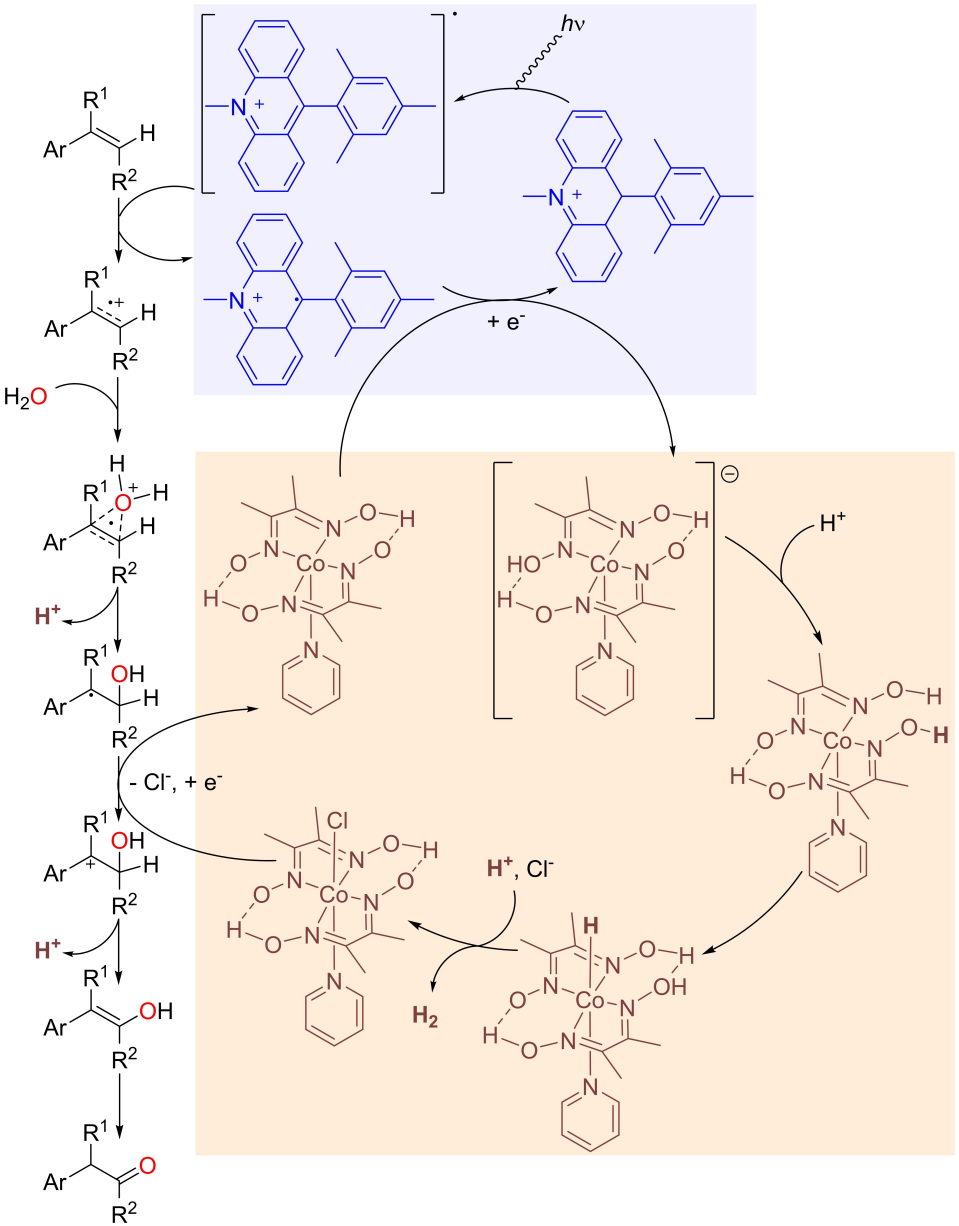
Postulated reaction mechanism of the photocatalytic anti‐Markovnikov Wacker‐type oxidation of β‐alkyl styrenes by water with the liberation of dihydrogen.

Dual catalysis has emerged in the last decades as a powerful approach to generate sustainable processes and most importantly, to access fundamentally new transformations.^[36]^ In particular, the combination of transition metal catalysts and photochemical processes has gained tremendous interest nowadays.^[37]^ In this respect, Lei and co‐workers reported a photocatalytic anti‐Markovnikov oxidation of olefins with the participation of a cobalt‐based proton‐reducing catalyst, namely Co(dmgH)_2_pyCl (dmgH=dimethylglyoximate monoanion; py=pyridine).^[38]^ The reaction design considers water as the source of oxygen, and dihydrogen is liberated with a perfect atom economy (Scheme 18). The reactions were conducted in acetonitrile solution under irradiation with blue LEDs with an acridinium photosensitizer at room temperature for 24 h. The catalysis was applicable only to aromatic olefins, since the aliphatic ones were unreactive due to their high oxidation potential. A catalytic cycle was proposed based on labeling studies, kinetic isotopic effects, and CV analysis (Scheme 19). The photosensitizer generates a radical cation in the olefin that undergoes an anti‐Markovnikov addition of water with a deprotonation that leads to a carbocation after oxidization with the cobalt complex. The ketone is formed after proton elimination and the corresponding keto–enol tautomerism. H_2_ is produced in the cobalt catalytic cycle, while the photocatalytic form of acridinium is regenerated. It is noteworthy that the Milstein group reported a procedure for the Markovnikov oxidation of styrenes by water with H_2_ liberation by using a noble‐metal ruthenium complex as a precatalyst in the presence of a Lewis acid.^[39]^

### 3.2 Wacker‐Type Reactions with Copper‐Based Catalysts

**Scheme 20:**
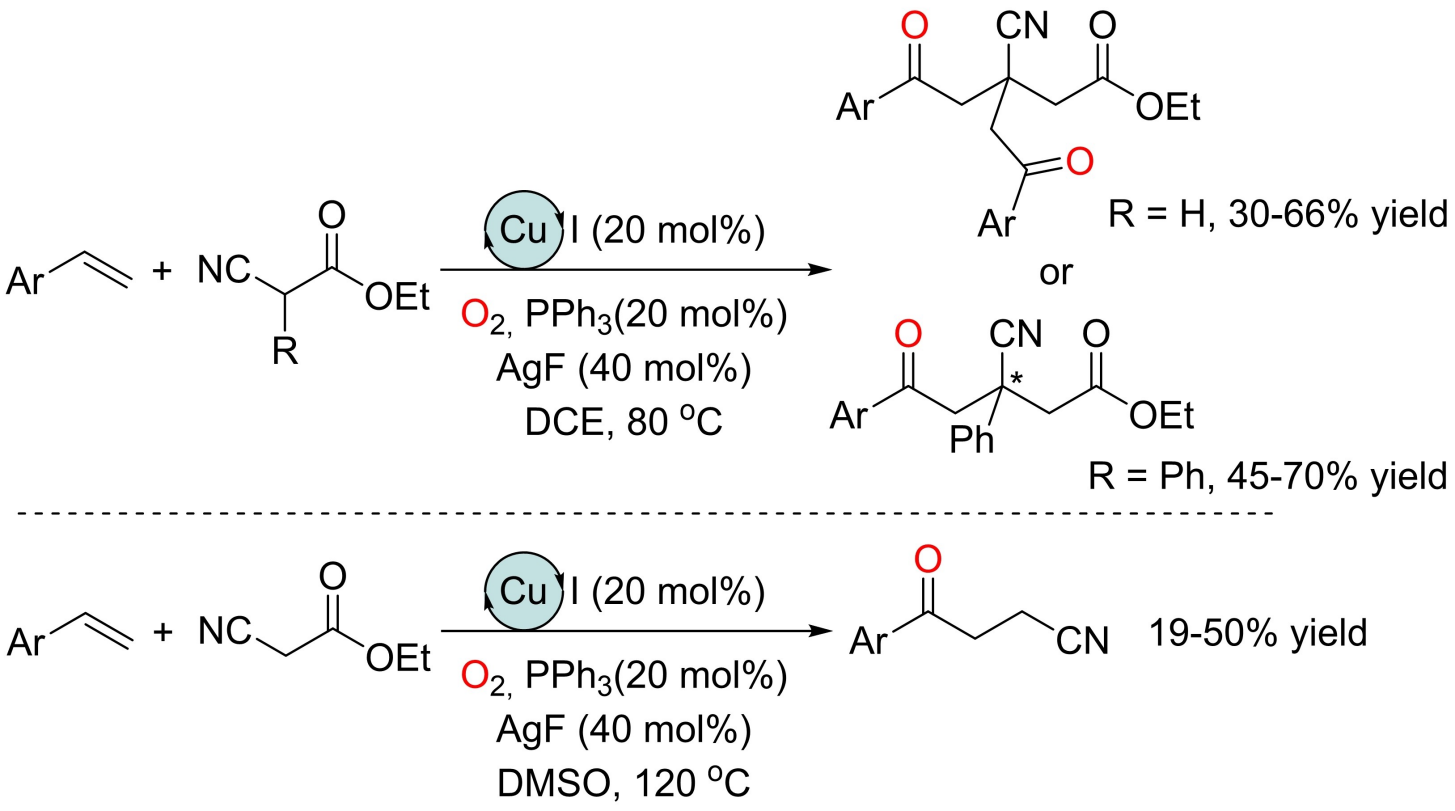
Simultaneous copper‐catalyzed Wacker‐type oxidation of styrenes and enolate cross‐coupling with cyanoesters in an O_2_ atmosphere at high temperatures.

A unique copper‐catalyzed oxidation of olefins was reported by Zhang *et al*. during a multicomponent transformation.^[40]^ Specifically, α‐cyanoesters reacted with styrenes in the presence of air to access γ‐ketoesters as a result of mono‐ or bis α‐enolation (Scheme 20, top). Alternatively, γ‐ketonitriles were formed in a chemoselective manner and short reaction time by changing the solvent and increasing the reaction temperature (Scheme 20, bottom). These transformations might be regarded as an oxidative enolate–olefin cross‐coupling. CuI was identified as the optimal precatalyst in the presence of a triphenylphosphine (PPh_3_) ligand and sub‐stoichiometric amounts of AgF, whose role was attributed to be as a co‐oxydant. However, one can speculate that silver could also play the role of iodide scavenger to generate cationic copper species, which are typically more active than the neutral ones.^[41]^ The γ‐ketoester products bear a stereogenic quaternary α‐center, which paves the way for the search for asymmetric variants.

**Scheme 21:**
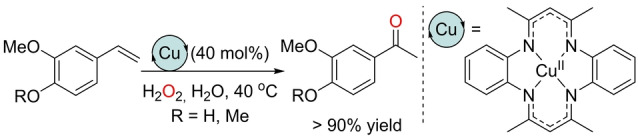
Copper‐catalyzed Wacker‐type oxidation of styrenes to ketones with H_2_O_2_ as the oxidant in water.

All these above‐stated examples demonstrate the ability of Fe, Co, and Cu complexes to be engaged as homogeneous catalysts in the Wacker‐type oxidation of olefins. They share in common the need to use chlorinated or alcohol solvents as well as different types of additives. Consequently, the quest for more benign reaction conditions has also been explored. Using water as the media and/or reagent is clearly an appealing as well as a challenging approach to bring Wacker‐type reactions close to the principles of green chemistry.^[42]^ In this context, a first contribution was reported in 2017 by the Meng group, who developed a copper‐catalyzed Wacker‐type oxidation of styrenes to ketones in water with H_2_O_2_ as the oxidant (Scheme 21).^[43]^ A high catalyst loading was required and no rational or mechanistic studies were performed to understand the exact role of the catalyst.

**Scheme 22:**
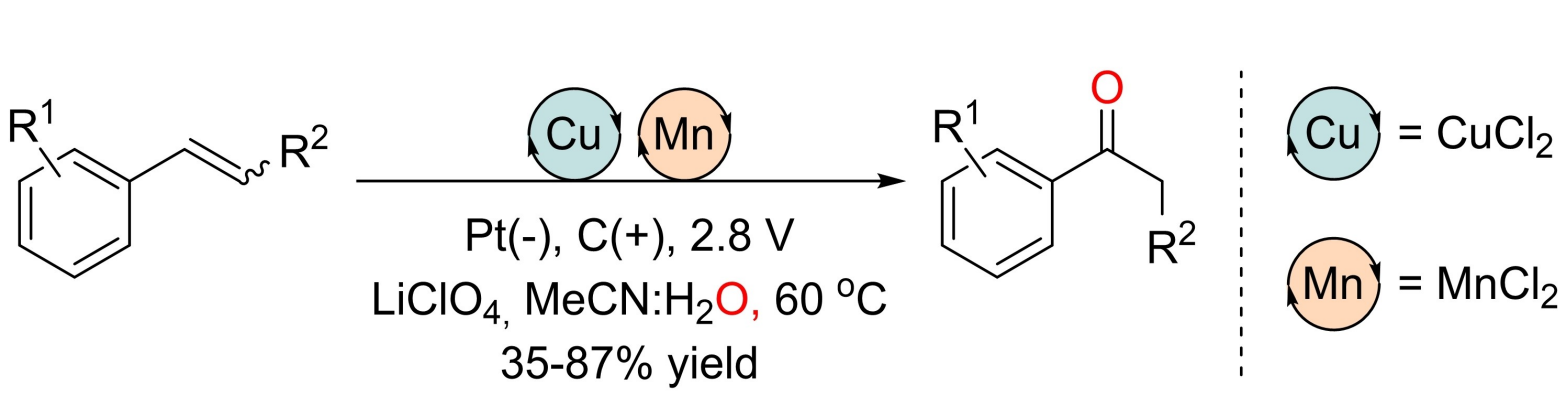
Mn/Cu co‐catalyzed electrochemical Wacker‐type oxidation of styrenes without any external chemical oxidant in aqueous media.

**Scheme 23:**
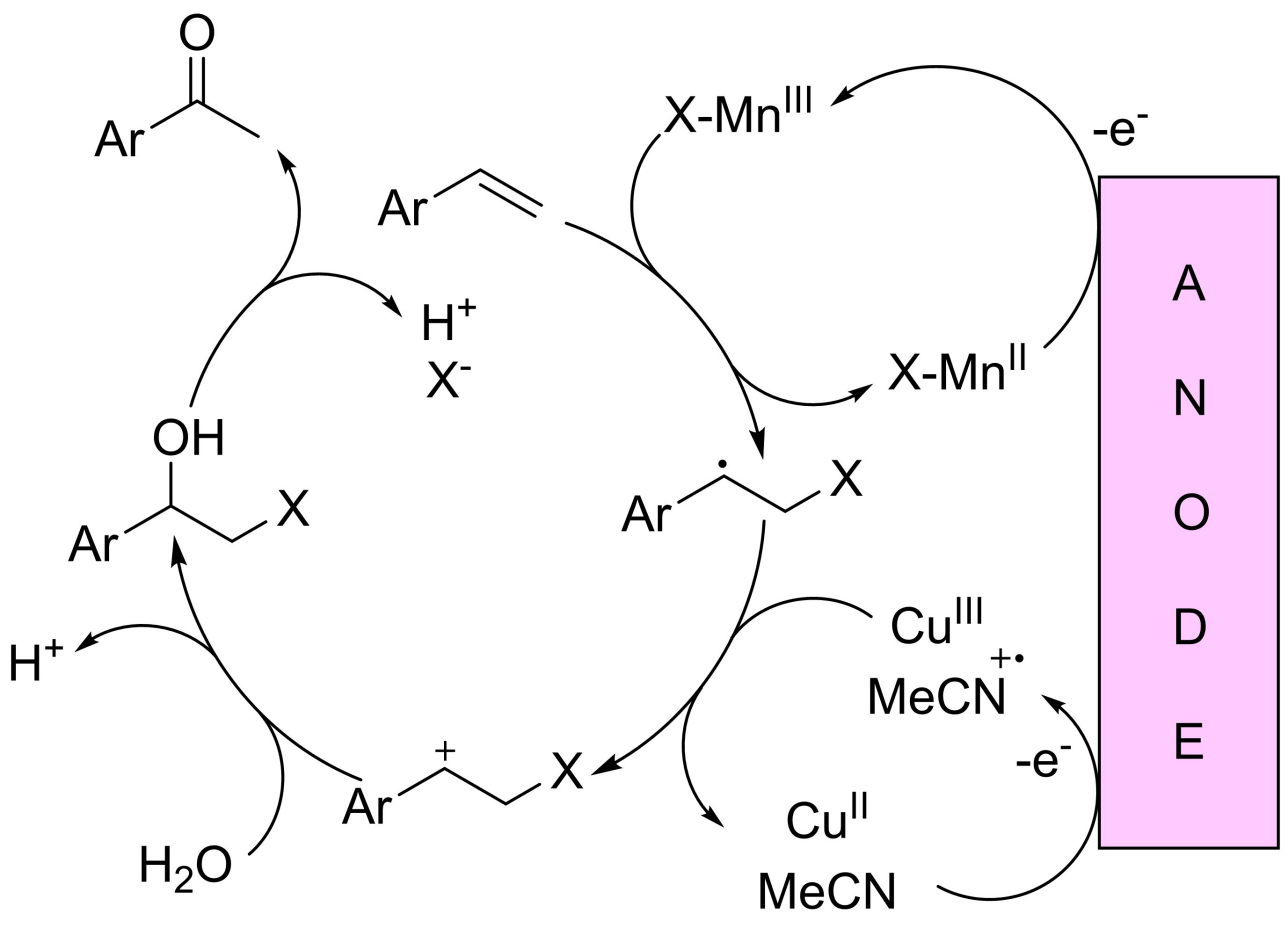
Postulated reaction mechanism for the Cu/Mn co‐catalyzed electrochemical Wacker‐type oxidation of styrenes. The elementary steps related to the anode are shown; the reduction of protons to H_2_ occurs at the cathode. X=Cl or Br.

In 2020, Lai and Pericàs reported an electrochemical Wacker‐type reaction in the presence of catalytic amounts of both copper and manganese salts, with water being the source of oxygen in the absence of any external chemical oxidant (Scheme 22).^[44]^ The reactions were suitable for styrenes, with excellent Markovnikov selectivity to deliver the corresponding ketones at 60 °C. The procedure was carried out in an undivided cell at a constant potential of 2.8 V, with acetonitrile as a co‐solvent and LiClO_4_ salt. From a mechanistic point of view, several control experiments support the formation of several redox processes, as depicted in Scheme 23. First, the electrochemically formed Mn^III^ halide transfers a halogen atom to the terminal olefin, thereby leading to a radical intermediate that undergoes oxidation to a carbocation by an *in situ* electrogenerated Cu^III^ and/or acetonitrile radical cation. After hydration, halohydrin is formed, followed by dehydrohalogenation to generate the ketone product through keto–enol tautomerism. Note that all these redox events take place at the anode, whereas at the cathode, protons are reduced to dihydrogen (H_2_). This contribution shows one of the possible advantages of combining two different transition metals from the first row of the periodic table.

### 3.3 Wacker‐Type Reactions with Nickel‐Based Catalysts

**Scheme 24:**
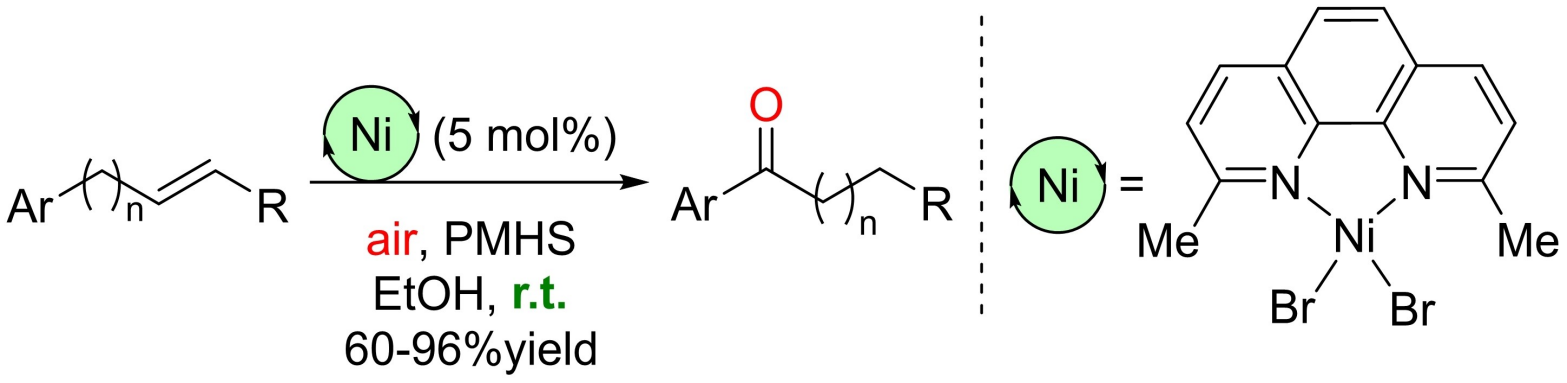
Aerobic nickel‐catalyzed remote Wacker‐type oxidation of olefins at room temperature.

**Scheme 25:**
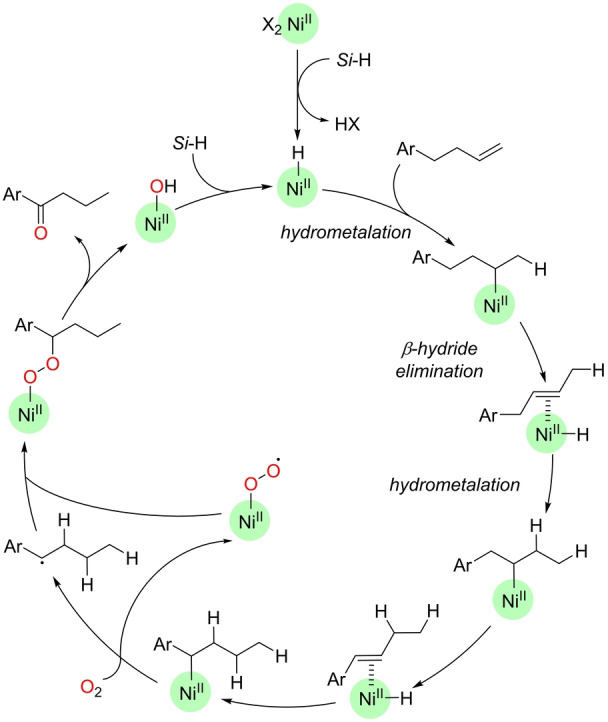
Postulated reaction mechanism of the aerobic nickel‐catalyzed remote Wacker‐type oxidation of olefins by exploiting a chain‐walking mechanism (the neocuproine ligand around the nickel center is not depicted for the sake of clarity). X=Br or OEt, *Si*‐H=PMHS or other hydrosilane.

The Han group reported in 2019 the only known example of a nickel‐catalyzed Wacker‐type oxidation (Scheme 24).^[45]^ Starting from internal aliphatic olefins, the reaction design aimed at forming catalytically active nickel‐hydride species that undergo a chain‐walking mechanism (iterative hydrometalation and β‐hydride elimination by nickel; Scheme 25) prior to a nickel(II)‐catalyzed oxidation with Markovnikov selectivity. In this case, the catalyst precursor is formed by a combination of a NiBr_2_ complex and neocuproine ligand. PMHS hydrosilane is used as both the reducing agent and the source of hydrogen atoms in the final product. The catalysis takes place at room temperature in an air atmosphere, with ethanol being the solvent of choice. Although the reaction is limited in scope to substrates containing aromatic motifs that stabilize the final intermediate from the nickel‐catalyzed chain‐walking mechanism (Scheme 25), this transformation shows that nickel catalysts promote Wacker‐type oxidations and that they can be employed in one‐pot multistep sequences, which is relevant in the context of sustainability.^[46]^

## 4 Wacker‐Type Reactions in the Absence of Metal Catalysts

**Scheme 26:**
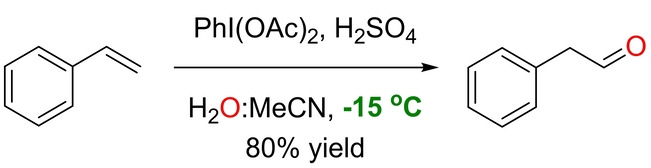
Metal‐free oxidation of styrene to acetaldehyde in the presence of the PhI(OAc)_2_ reagent.

Avoiding any metal species in Wacker‐type oxidations of olefins remained a challenge until the beginning of the 21st century. In two seminal contributions, Yusubov et al. showed the ability of the hypervalent iodine compound PhI(OAc)_2_ to react with styrene to form phenylacetaldehyde in the absence of any metal species (Scheme 26).^[47]^ The product resulted from an anti‐Markovnikov selectivity‐determining step. The reaction proceeded at −15 °C in the presence of the strong Brønsted acid H_2_SO_4_ and a mixture of solvents (H_2_O and MeCN). The role of iodonium species as the oxidating species was evoked,^[48]^ but the exact reaction mechanism was not studied.

**Scheme 27:**
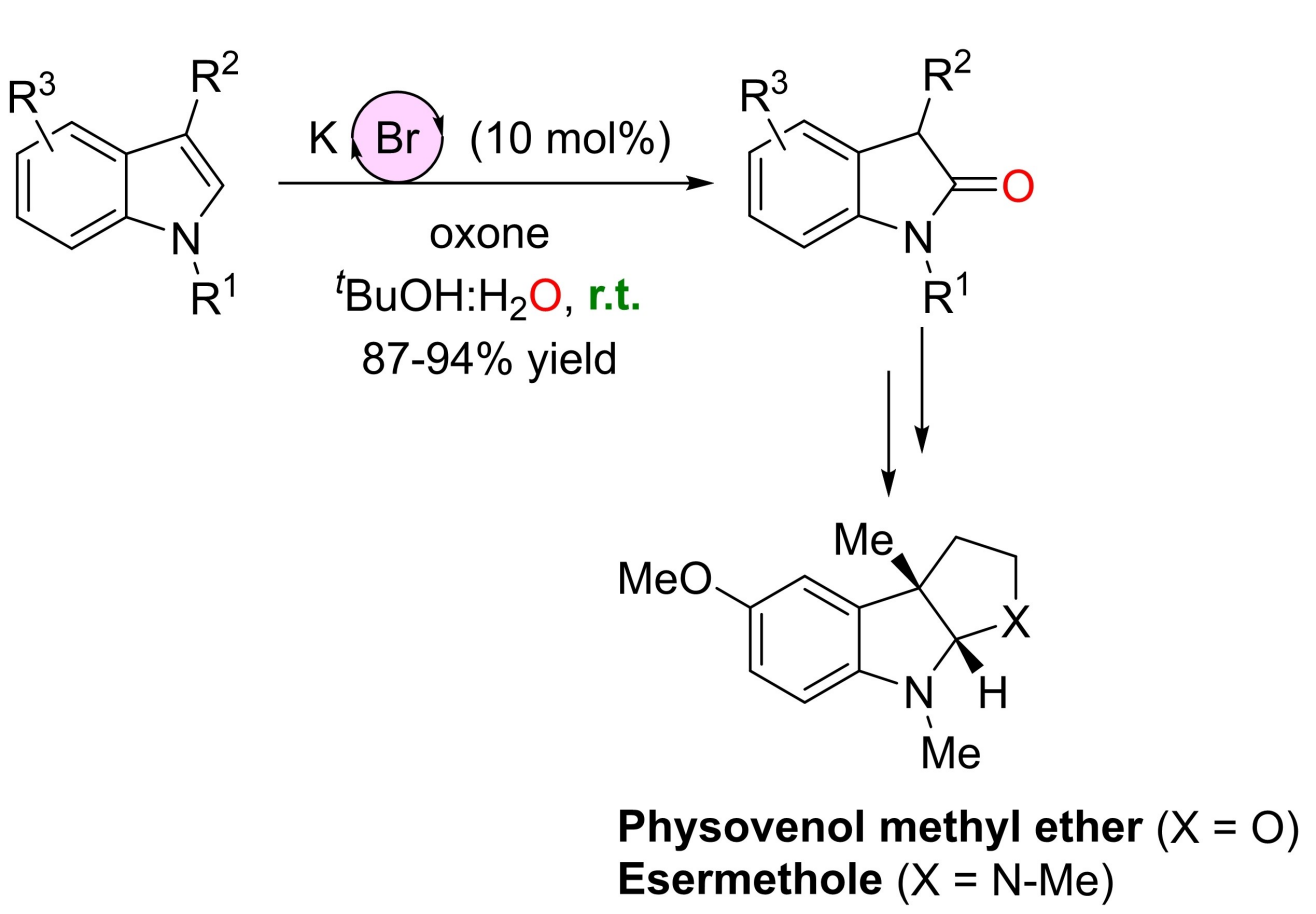
Oxidation of indoles to 2‐oxindoles in the presence of catalytic amounts of KBr assisted by oxone at room temperature, and its application in the total synthesis of two natural products.

**Scheme 28:**
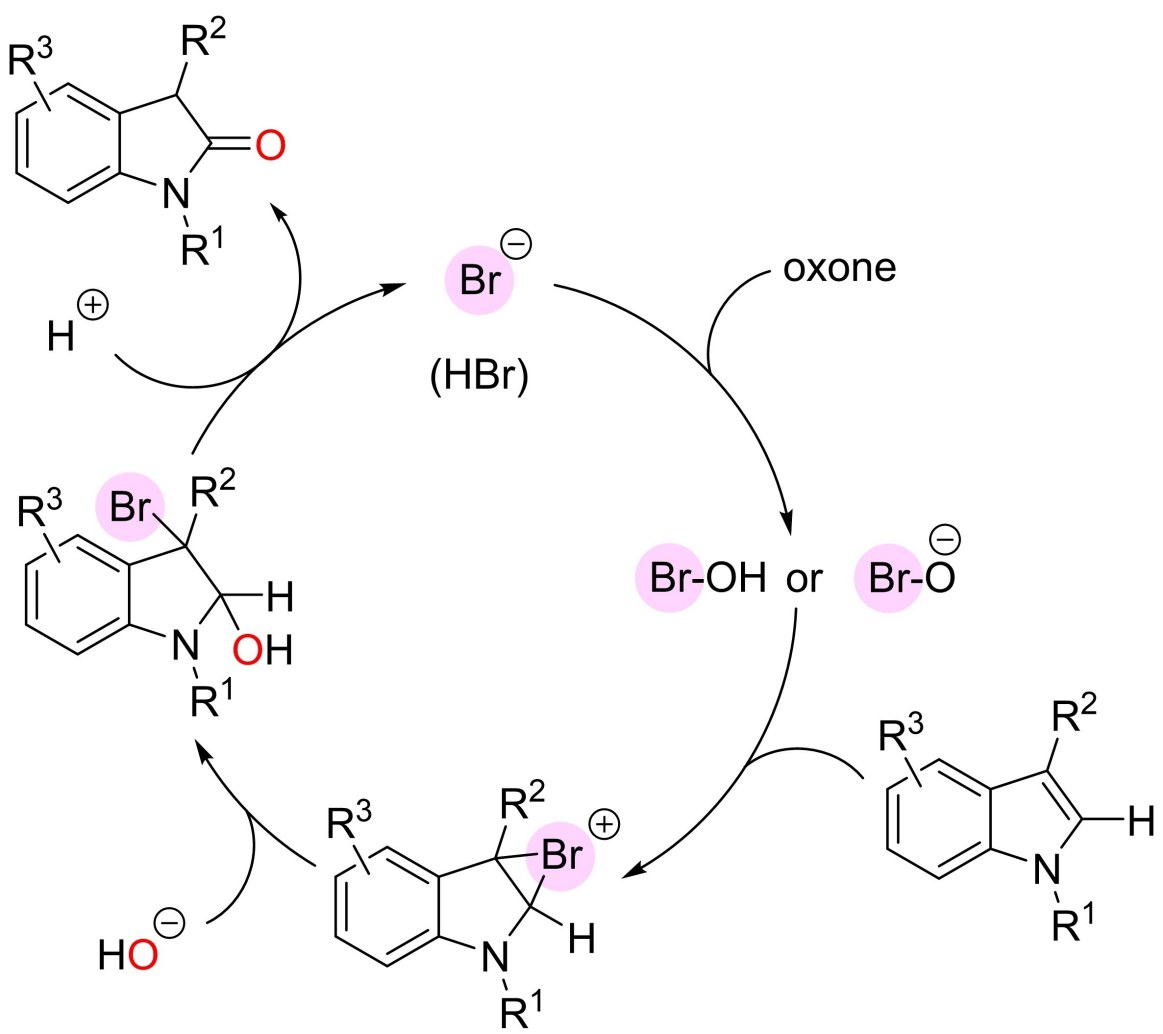
Proposed reaction mechanism of the oxidation of indoles to 2‐oxindoles in the presence of catalytic amounts of KBr assisted by oxone.

Considering the growing interest in developing metal‐free catalysts, for which halide(III) species occupy a place of choice,^[49]^ Tong and co‐workers developed a method for the oxidation at room temperature of the olefinic bond of indoles by catalytically forming bromonium species provided that oxone (potassium peroxymonosulfate) was employed as a stoichiometric oxidant (Scheme 27).^[50]^ Investigation of the reaction identified KBr as the source of the bromide ions and *t*BuOH/water as the solvents. The substrate scope was restricted to 3‐alkyl‐substituted indoles, which furnished the corresponding 2‐oxindoles. Many functional groups were tolerated in different positions of the backbone, including the challenging free N−H indoles. The applicability of this method was demonstrated in the total synthesis of two natural products: Physovenol (isolated as the methyl ether derivative) and Esermethole (Scheme 27). From a mechanistic point of view, bromide firstly reacts with oxone to form hypobromite, which reacts with the olefin to give a cyclic bromonium cation that undergoes ring opening by nucleophilic attack of a hydroxy group (Scheme 28). Debromination in the resulting intermediate affords the ketone product and oxone further converts the remaining bromide anion into the catalytically active hypobromite species (Scheme 28).

**Scheme 29:**
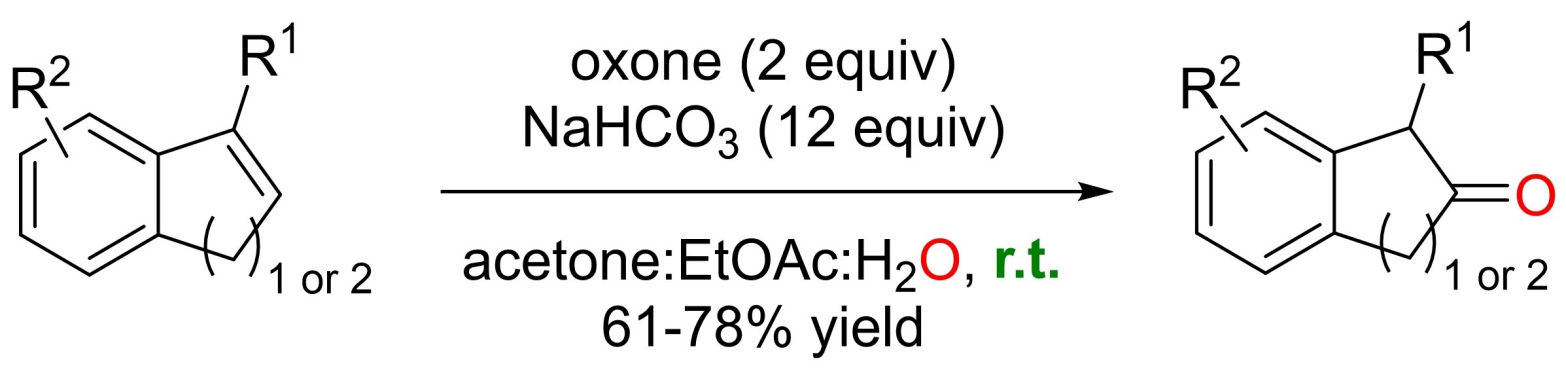
Anti‐Markovnikov‐selective oxidation of indene and 1,2‐dihydronaphthalene derivatives mediated by oxone at room temperature.

**Scheme 30:**
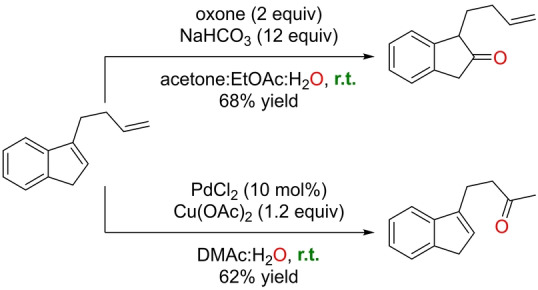
Comparison of site‐selective oxidations mediated by oxone (top) *versus* a palladium catalyst (bottom) for an allyl‐containing indene substrate at room temperature. DMAc=*N*,*N*‐dimethylacetamide.

In the previous example, oxone was employed as the oxidizing agent to generate the catalytically productive halonium species. Alternatively, Phatake and Ramana described an oxidation of olefins to ketones that employs oxone as the single reagent.^[51]^ The method, which is based on the use of NaHCO_3_ as an additive in large amounts and a ternary mixture of solvents (acetone, ethyl acetate, and water), is compatible with indenes and 1,2‐dihydronapthalenes, thereby affording the anti‐Markovnikov products 2‐indanones and 2‐tetralones, respectively (Scheme 29). This procedure reversed the site‐selectivity encountered in the classical palladium‐catalyzed Wacker‐type Markovnikov oxidation of an allyl‐containing indene substrate (Scheme 30). The current procedure oxidized the indene core in a selective manner to leave the allyl group unreacted, whereas the palladium‐catalyzed process oxidized the allyl moiety, keeping the indene core intact. Preliminary investigations indicate that neither the epoxide nor the acetonide are involved in the mechanism, and the presence of trace impurities of metals was ruled out by a metal scavenger test. However, the overall mechanism of this reaction remains to be determined. Compared to metal‐based catalysts, the current metal‐free systems for Wacker‐type reactions display lower functional group tolerance because of the strong oxidizing power associated with the reagents.

## 5 Conclusions and Perspectives

Since the first report on Wacker‐type oxidation of olefins more than 60 years ago, such transformations have stimulated the creativity of chemists to design more sustainable procedures. As a result of its atom‐economic nature and the highly value‐added carbonyl‐containing products formed, tremendous efforts have been devoted to develop regioselective catalysts. In addition to the notable breakthroughs reached by noble palladium catalysts,^[52]^ recent advances have shown the ability of first row transition metals to replace the scarce and costly palladium, while still affording excellent levels of reactivity with absolute control of the regioselectivity. This is particularly true for aromatic olefins and less pronounced for terminal aliphatic olefins, not to mention the challenge of internal aliphatic olefins, in which a statistical mixture of ketones can form. Moreover, mild reaction conditions have been developed by avoiding the use of both a copper co‐catalyst and strongly acidic media to make the catalysis compatible with sensitive functional groups, which is relevant for the synthesis of fine‐chemicals. In addition, reducing the reaction temperature to 20–30 °C and replacing high pressures of O_2_ by ambient air makes these methods attractive for implementation in any type of laboratory.

The most widely exploited metal to replace palladium in Wacker‐type oxidations is iron, and the efficiency of these methods is exemplified by its application in the total synthesis of natural products. All the examples described above highlight that the steric and electronic effects of the ligands attached to the metal center play a key role in controlling the reaction coordinate towards the selective formation of the Markovnikov or anti‐Markovnikov carbonyl products. Most of the catalysts based on first row transition metal catalysts concern purely homogeneous systems, although promising results have been obtained with heterogeneous and biocatalysts, especially for asymmetric versions. A common feature of the first‐row catalysts is that they can stabilize metal‐oxo and metal‐peroxo species as well as metal‐hydride species that facilitate the formation of metal‐alkyl intermediates. Such mechanistic considerations are different from those established for palladium catalysts in Wacker‐type reactions and they complement each other. The study in more detail of the plausible elementary steps of the catalytic cycles should translate into the design of more powerful catalysts in the near future. It might even be reasoned that such a field is still in its infancy when considering the many other first‐row transition metal catalysts that could be envisaged. Interestingly, the conceptualization of reaction designs considering cooperative mechanisms, electrocatalysis, photocatalysis, and hypervalent halides should enlarge the possibilities to provide ketones (or aldehydes) from olefins in a more benign manner.

## Conflict of interest

The authors declare no conflict of interest.

## Biographical Information

*Purushothaman Rajeshwaran obtained his BSc and MSc in Chemical Sciences at Vellore Institute of Technology (India) under the supervision of Prof. Thirumanavelan Gandhi. In 2021 he joined the International Master in Molecular Catalysis and Green Chemistry at the University of Rennes (France) and he is currently carrying out his MSc internship under the supervision of Dr. Rafael Gramage‐Doria, where he focuses on supramolecular iridium catalysis*.

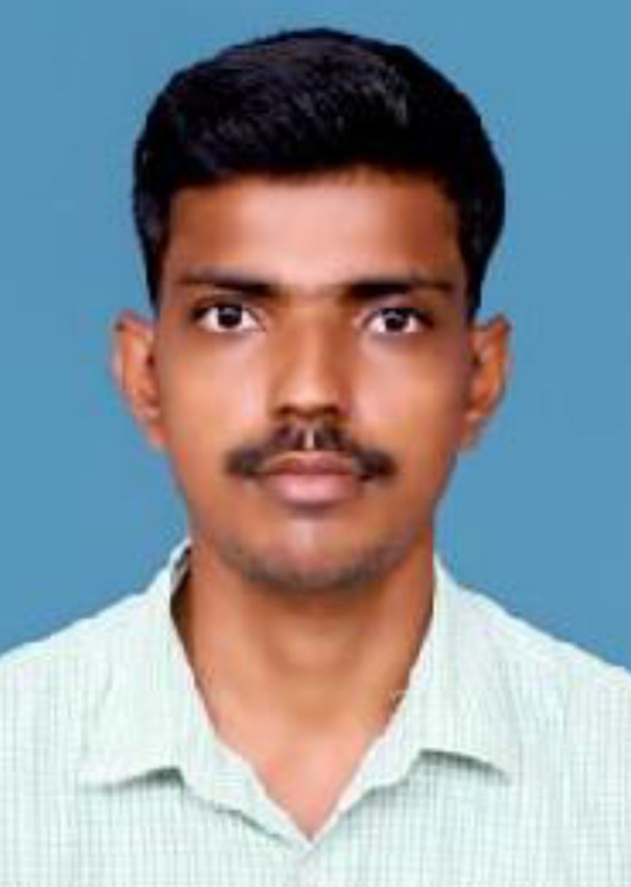

## Biographical Information

*Jonathan Trouvé obtained his MSc in Organic Chemistry from the University of Caen (France) in 2019 under the supervision of Dr. Bénédicte Lepoittevin and Dr. Jerôme Baudoux, with a research project on zwitterionic polymers. Currently, he is pursuing his PhD under the supervision of Dr. Rafael Gramage‐Doria at the University of Rennes (France), where he focuses on supramolecular and bio‐inspired transition metal catalysis*.

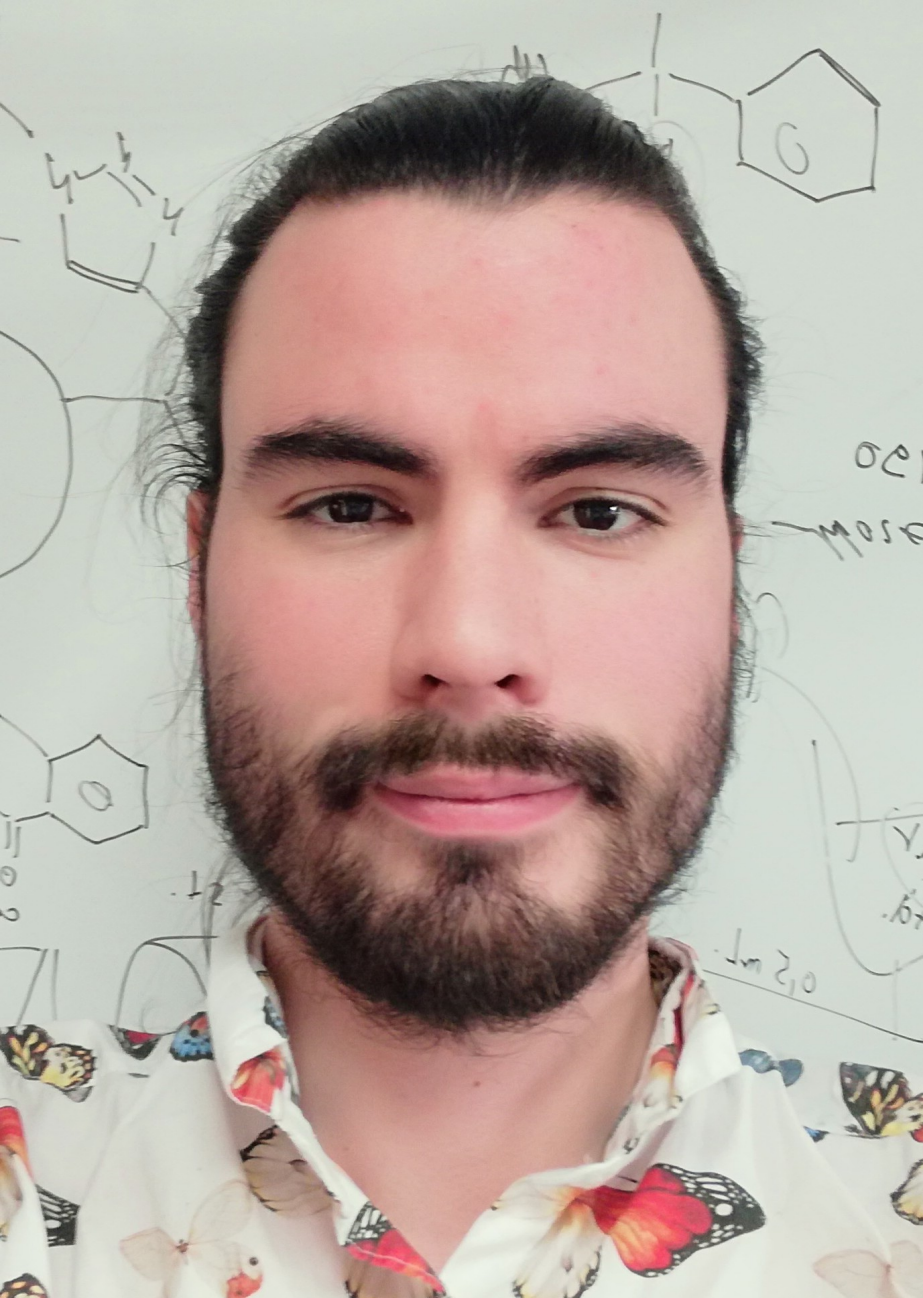

## Biographical Information

*Khalil Youssef obtained his BSc in Chemical Sciences in Lebanon and joined the International Master in Molecular Catalysis and Green Chemistry at the University of Rennes (France) in 2019. He completed his MSc internship under the supervision of Dr. Rafael Gramage‐Doria, where he focused on bio‐inspired transition metal catalysis. Currently, he is pursuing his PhD under the supervision of Prof. Dominique Lorcy at the same university*.

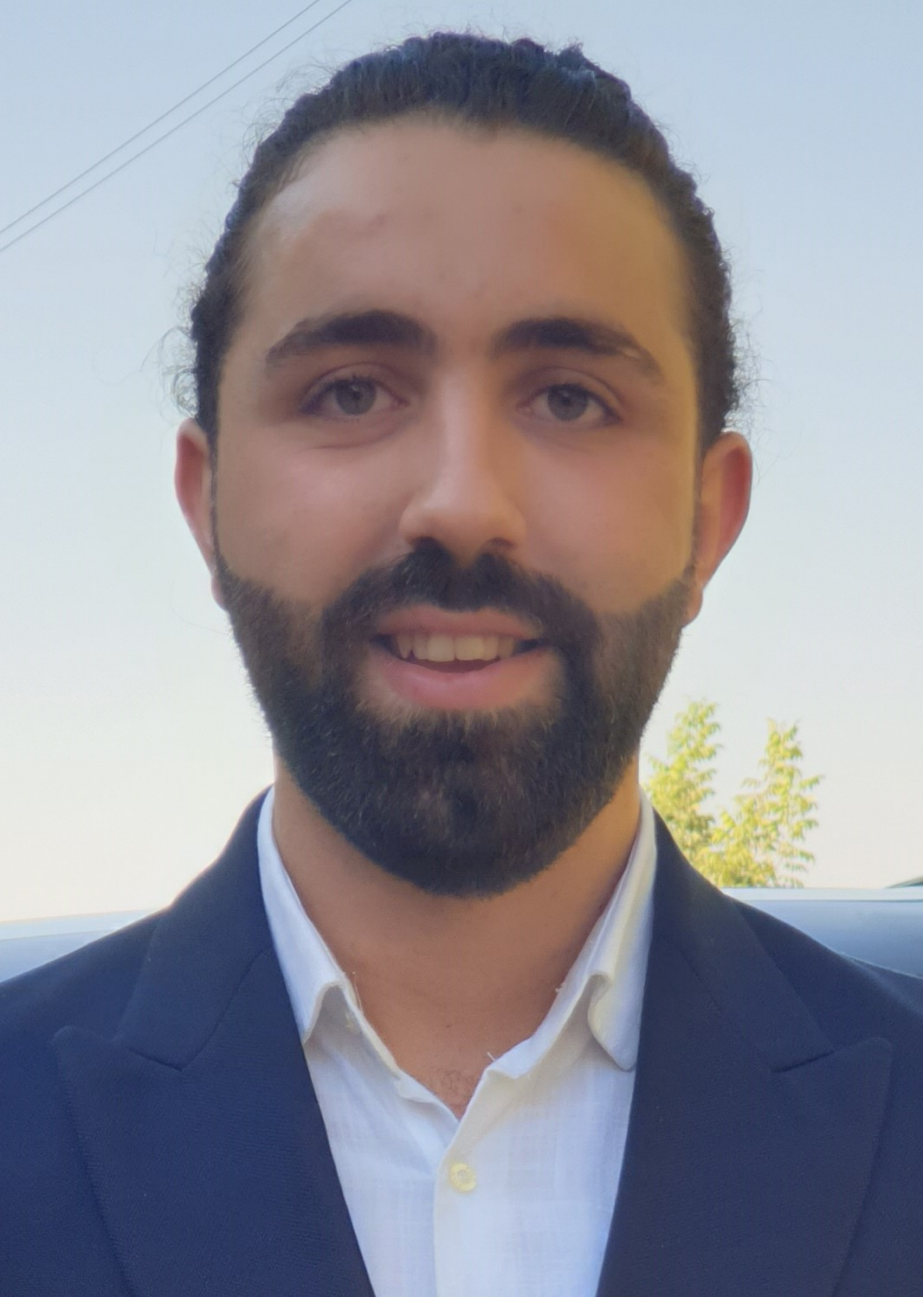

## Biographical Information

*Rafael Gramage‐Doria received his PhD from the University of Strasbourg (France) with Dr. Dominique Matt and Prof. Dominique Armspach. After postdoctoral fellowships with Prof. Joost N. H. Reek at the University of Amsterdam (Netherlands) and Prof. Takashi Ooi at Nagoya University (Japan), he joined in 2015 the Institute of Chemical Sciences of the University of Rennes (France) as a CNRS researcher, where he completed his Habilitation (2019). His research includes transition metal catalysis for fine‐chemicals and green chemistry applications, C−H bond functionalization, coordination chemistry, and supramolecular and bio‐inspired catalysis*.